# Developmental disruptions underlying brain abnormalities in ciliopathies

**DOI:** 10.1038/ncomms8857

**Published:** 2015-07-24

**Authors:** Jiami Guo, Holden Higginbotham, Jingjun Li, Jackie Nichols, Josua Hirt, Vladimir Ghukasyan, E.S. Anton

**Affiliations:** 1 UNC Neuroscience Center and the Department of Cell Biology and Physiology, University of North Carolina School of Medicine, Chapel Hill, North Carolina 27599, USA

## Abstract

Primary cilia are essential conveyors of signals underlying major cell functions. Cerebral cortical progenitors and neurons have a primary cilium. The significance of cilia function for brain development and function is evident in the plethora of developmental brain disorders associated with human ciliopathies. Nevertheless, the role of primary cilia function in corticogenesis remains largely unknown. Here we delineate the functions of primary cilia in the construction of cerebral cortex and their relevance to ciliopathies, using an shRNA library targeting ciliopathy genes known to cause brain disorders, but whose roles in brain development are unclear. We used the library to query how ciliopathy genes affect distinct stages of mouse cortical development, in particular neural progenitor development, neuronal migration, neuronal differentiation and early neuronal connectivity. Our results define the developmental functions of ciliopathy genes and delineate disrupted developmental events that are integrally related to the emergence of brain abnormalities in ciliopathies.

## Abstract (web summary)

Primary cilia are essential conveyors of signals underlying major cellular functions but their role in brain development is not completely understood. Here the authors compiled a shRNA library targeting ciliopathy genes known to cause brain disorders, and used it to query how ciliopathy genes affect distinct stages of mouse cortical development.

## Graphical abstract

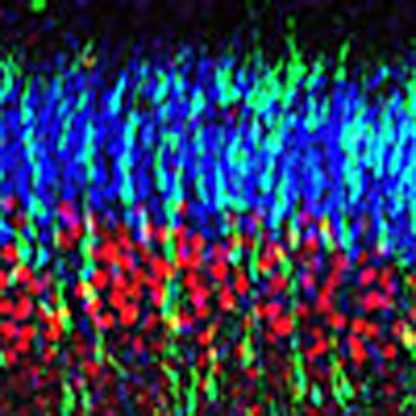

Cerebral cortex forms as a result of a coordinated sequence of events: radial progenitor formation, neurogenesis, neuronal migration, post-migratory neuronal differentiation and connectivity. An efficient intracellular response to extracellular signalling cues is fundamental for coordinating the various cellular events that underlie the formation of cerebral cortex. Primary cilia, the microtubule-based, slender, antenna-like projections from cells, are essential integrators and conveyors of signal transduction. Cortical neuronal progenitors and developing neurons have a primary cilium. The significance of cilia function for cortical development and function^1^ is evident in developmental brain disorders such as Joubert, Meckel–Gruber, orofaciodigital and Bardet–Biedl syndromes (commonly referred to as ciliopathies), where disrupted cilia function and the resultant changes in cortical formation underlie cognitive deficits and intellectual disabilities^2^,^3^,^4^. Moreover, neuropsychiatric disorders such as autism spectrum disorders and schizophrenia are also associated with human ciliopathies^2^,^3^,^4^,^5^. These observations suggest that impaired cilia function can hinder the development of neural circuitry and activity, leading to significant functional deficits. Therefore, delineating specific functions of ciliopathy-associated genes in distinct stages of cortical development can help decipher the biological basis of brain function abnormalities in ciliopathies.

Functional genomic analysis of a cilium has led to the identification of a diverse group of cilia and centrosome-specific proteins^1^,^2^,^3^,^4^,^6^,^7^,^8^. Among them, mutations in 87 of these genes have been associated with human ciliopathies (Supplementary Tables 1 and 2); mutations in 77 of these genes thus far are known to result in neurobehavioural or neurodevelopmental deficits in humans (Supplementary Tables 1–3). A necessary first step in our ability to devise effective therapeutic interventions for these cilia-related neurodevelopmental diseases is an understanding of their biological basis. Disrupted neural circuitry due to genetic mutations in cilia-specific genes in ciliopathy patients could arise as a result of deficits in the generation of appropriate number and types of neurons, disrupted neuronal migration, placement or connectivity in the developing brain. Pinpointing which of these processes is disrupted as a result of a genetic mutation can be a complex and time-consuming process. However, a short hairpin RNA (shRNA) library-based gene knockdown approach coupled with routinized assays for distinct stages of cortical development allow for rapid and efficient assessment of the brain developmental roles of ciliopathy genes. With this goal in mind, we compiled a library of shRNA targeting 30 genes known to be linked to human ciliopathies with neurological deficits (Supplementary Table 4). We used this library to query how ciliopathy-related genes affect distinct stages of brain development, in particular neural progenitor development, neuronal migration, neuronal differentiation and early neuronal connectivity, following shRNA-mediated gene knockdown or control shRNA expression in embryonic brains. These studies give us a comprehensive insight into how human ciliopathy-related genes affect distinct stages of cerebral cortical formation, and reveal hitherto undefined neurodevelopmental pathways whose disruption is likely to be integrally related to the development of neurological deficits in human ciliopathies.

## Results

### shRNA library of ciliopathy genes

Mutations in 87 cilia-related genes have thus far been associated with human ciliopathies (Supplementary Tables 1–3). Mutations in 77 of these genes are known to be associated with neurobehavioural or neurodevelopmental deficits in humans (Supplementary Tables 1–3). The developmental and neurological consequences of mutations associated with the majority of these genes are yet to be clarified. We compiled a library of shRNAs targeting 30 of the ciliopathy genes with defined links to neurodeficits in humans to systematically evaluate their function in cerebral cortical development. Previously well-studied ciliopathy-related genes with known developmental functions (for example, ARL13B^9^,^10^, INPP5E^11^,^12^ and IFT88 (ref. 13)) were excluded. This library was assembled using the resources available at the UNC Gene Therapy Center (Supplementary Table 4). Three to six different shRNAs for each gene were combined into two pools to achieve knockdown efficiency (KE). Effects were confirmed with duplicated pools. As control for off-target disruptions, a vector containing scrambled shRNA was used. Target gene KE of shRNAs was validated using quantitative reverse transcription–PCR (qRT–PCR) (Supplementary Table 5). Validity of specific shRNAs was further checked by randomly analysing off-target effects on other ciliopathy genes in the list. No such off-target knockdown was detected. Further, several shRNAs were found not to induce expected changes in specific gene expression. They were used as additional controls and did not produce any changes in our cortical development assays.

### shRNA library screen for ciliopathy gene functions in brain

To understand how ciliopathy-related genes affect early brain development, different ciliopathy gene-specific shRNA or control shRNA vectors were electroporated into the developing cerebral cortex at E14.5. For some of the genes (*BBS7*, *BUBR1* and *KIF7*), rescue of shRNA effects was tested using co-electroporation of shRNA-resistant human complementary DNAs (cDNAs) (UNC Gene Therapy Center). Electroporated embryos were allowed to survive for 2 or 4 days *in vivo*, and were analysed for progenitor proliferation/organization, neuronal migration, laminar organization and differentiation of post-migratory cortical neurons and their projections. The effect of shRNAs on distinct aspects of cortical development were assayed as follows.

**Figure 1:**
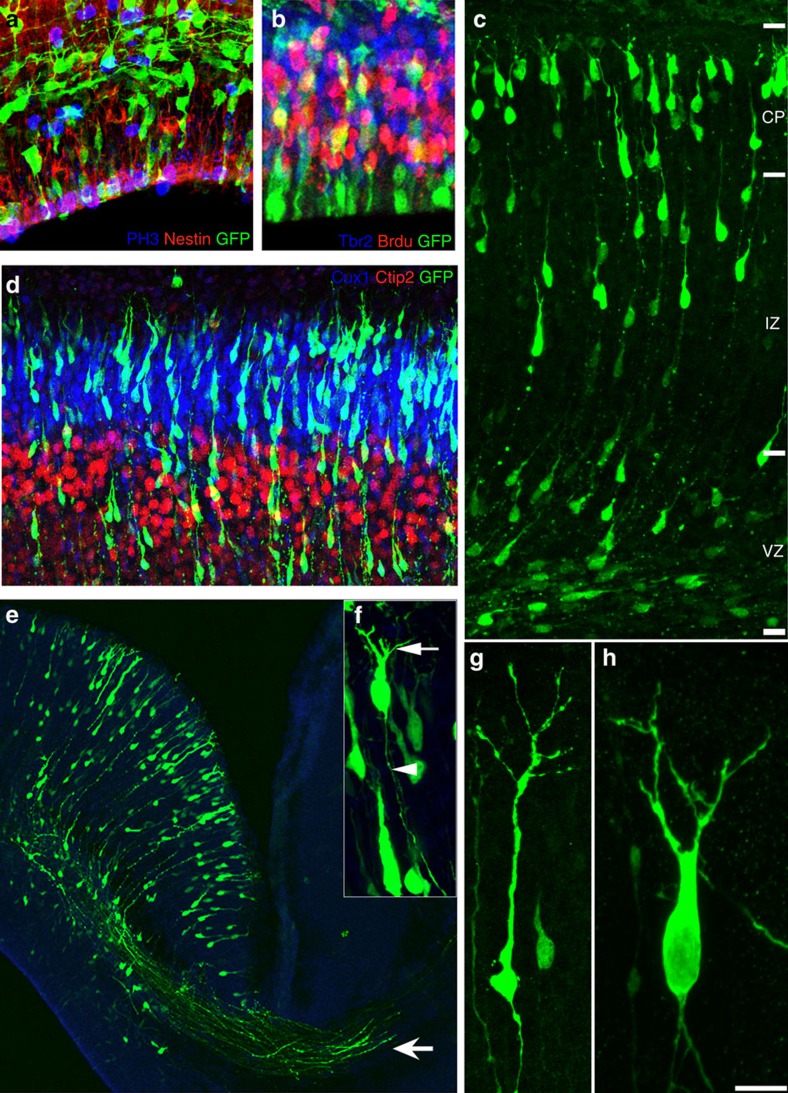
*In utero* electroporation-based assays for distinct stages of cortical development. (**a**,**b**) Assays for cortical progenitor proliferation. shRNA-electroporated E14 embryos were allowed to survive for 2 or 4 days and shRNA-expressing (GFP^+^) radial progenitors were co-labelled with anti-nestin (red) and mitotic marker anti-PH3 (blue) antibodies (**a**). Similarly, actively proliferating intermediate progenitors were co-labelled with anti-Tbr2 (blue) and BrdU (red) in **b**. Changes in proliferation patterns of shRNA-expressing cells (GFP^+^) can be monitored in such assays. (**c**) Assay for neuronal migration. At 2 days after electroporation, the extent of migration of GFP^+^ shRNA-expressing neurons away from the ventricular zone (VZ) towards the cortical plate (CP) can be observed and measured in this assay. (**d**) The laminar identity of shRNA-expressing neurons in the cortical plate can be evaluated by co-labelling with different layer-specific markers. Cortical plate neurons in **d** were co-labelled with anti-Cux-1 (layer 2/3) and anti-Ctip2 (layer 5). (**e**) At E18.5, projections of the post-migratory cortical neurons to the contralateral cortex can also be visualized (arrow, **e**). In higher-magnification images of the cortical plate (**f**), the growing axon (arrowhead) and dendrites (arrow) of post-migratory neurons in the emerging cortical plate can be clearly visualized. (**g**,**h**) The developing dendritic morphology of two different post-migratory neurons. GFP^+^ cells in these images express control shRNA vector. The use of a ciliopathy gene shRNA library in such assays allow rapid evaluation of the role of ciliopathy-related genes in different aspects of cortical development. CP, cortical plate; IZ, intermediate zone; SVZ, subventricular zone; VZ, ventricular zone. Scale bar, 160 μm (**a**–**c**); 210 μm (**d**); 330 μm (**e**); 65 μm (**f**,**g**); 45 μm (**h**).

Cortical progenitors electroporated with shRNAs at E14.5 were labelled with radial glial (RG)-specific anti-RC2 or anti-Nestin antibodies or intermediate progenitor-specific anti-Tbr2 antibodies at E16.5. E16.5 embryos were pulse labelled with 5-bromodeoxyuridine (BrdU) 1 h prior to removal. RG morphology and the apical and basal endfeet of GFP^+^/RC2^+^ RG were examined for changes in RG polarity. The apical molecular polarity of RG was evaluated by the characteristic apical β-catenin enrichment at the ventricular surface. The RG progenitor division was evaluated using PH3 and BrdU immunolabelling. The number and position of GFP^+^/BrdU^+^ or PH3^+^ nuclei were used to examine changes in the rate of cell division and the position of progenitor nuclei during cell cycle. Actively proliferating intermediate progenitors were co-labelled with anti-Tbr2 and anti-BrdU antibodies to monitor the changes in their proliferation patterns (Fig. 1).

The extent of migration of newly generated GFP^+^ neurons was assessed by measuring the distance between ventricular surface and the leading front of GFP^+^ migratory neurons, and by binning the distribution of GFP^+^ neurons across the developing cortical wall. Directionality (polarity) of neuronal migration was categorized as ‘oriented’ or ‘misoriented’ based on the angle of orientation of the leading process of migrating neurons relative to the pial surface (oriented: 75°–90°, misoriented:<75°). The final positioning of cortical neurons within the developing cortical layers was investigated by co-labelling GFP^+^ neurons with antibodies to layer-specific markers Tbr1 (layer VI), Ctip2 (layer V) and Cux1 (layers II and III). Distribution of co-labelled neurons were quantified and compared between control and shRNA-expressing groups (Fig. 1).

Post-migratory differentiation of GFP^+^, shRNA-expressing neurons in the cortical plate (CP) was evaluated by determining whether these neurons display an axon, and if the axon projects first apically and then laterally towards contralateral cortical or subcortical regions. The elaboration of apical and basal neurites was evaluated by measuring the length and number of the primary neurite branches, and the density of filopodia on primary apical neurites (Fig. 1).

### The effect of ciliopathy-specific genes on cortical progenitors

**Figure 2:**
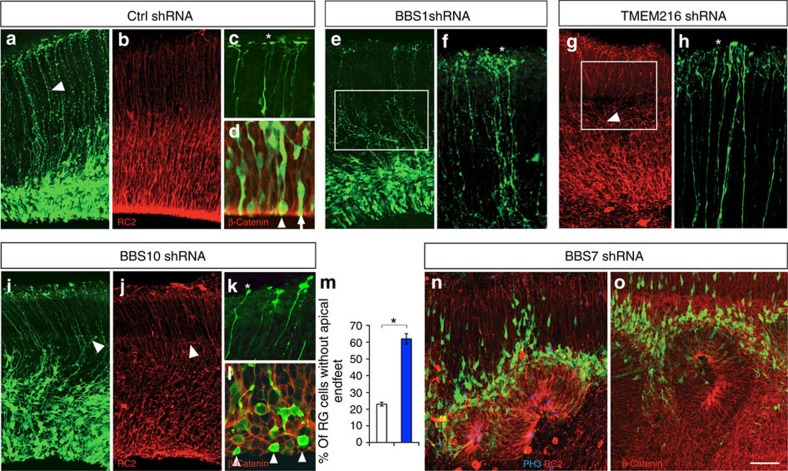
Ciliopathy-related gene effects on polarized radial glial progenitor morphology. (**a**–**d**) Control radial glial (RG) progenitor scaffold. Polarized RG are labelled with anti-GFP (**a**) and anti-RC2 (**b**). The basal processes of control radial glia (**a**, arrowhead) spanning the entire width of the developing embryonic cerebral cortices were labelled with electroporated GFP. (**c**) Higher-magnification view of basal endfeet (adjacent to asterisk) labelled with GFP. (**d**) Higher-magnification view of GFP^+^ radial glial cell bodies in VZ and apically enriched β-catenin (red) lining the ventricular surface. Radial glial cell soma is localized either adjacent to the ventricular surface (arrowhead) or just above with elongated apical endfeet (arrow). (**e**,**f**) BBS1 shRNA knockdown results in RG with wavy basal processes (square; **e**) and disrupted basal endfeet (see endfeet adjacent to asterisk; **f**). (**g**,**h**) TMEM216-deficient radial glia do not fasciculate as densely as in controls (square; **g**) and are often shorter and misoriented (arrowhead; **g**). (**h**) Disrupted basal endfeet in TMEM216 shRNA-expressing RG cells (see endfeet adjacent to asterisk). (**i**–**m**) Knockdown of BBS10 lead to aberrantly branched, short or retracted (arrowhead; **f**,**i**) RG scaffold. (**k**) Enlarged view of GFP^+^-expressing BBS10 shRNA basal endfeet illustrates the unbranched morphology of basal endfeet (see endfeet adjacent to asterisk). (**l**) BBS10 shRNA expression results in loss of apical endfeet, and apical enrichment of β-catenin (red). (**m**) Quantification of percentage BBS10 shRNA-expressing RG without apical endfeet. Number of cells: 134 (control); 189 (BBS10). (**n**,**o**) BBS7 shRNA expression results in rosette-like organization of the ventricular zone and aberrant β-catenin expression. Data shown are mean±s.e.m. *P<0.05 (Student’s *t*-test). Number of brains per group=4. Scale bar, 20 μm (**a**,**b**,**e**,**g**,**i**,**j**,**n**,**o**); 5 μm (**c**,**d**,**f**,**h**,**k**,**l**).

The generation and maintenance of the apicobasally polarized RG scaffold is an essential first step in cerebral cortical formation. Of the 30 ciliopathy genes tested, knockdown of four genes (*BBS1*, *BBS7*, *BBS10* and *TMEM216*) resulted in disruption of the apical–basal polarity of RG cells (Supplementary Table 6). Control RG progenitors display characteristic polarized morphology, with cell soma in the ventricular zone, an apical endfoot, an elongated polarized basal process oriented towards the pial surface and branched basal endfeet attached to the pial basement membrane (Fig. 2a–d). Knockdown of BBS1 (Bardet–Biedl syndrome 1) resulted in wavy RG processes (Fig. 2e) and excessively branched basal endfeet (Fig. 2f, asterisk). In contrast, TMEM216 (transmembrane protein 216) or BBS10 (Bardet–Biedl syndrome 10) deficiency led to short or retracted basal RG processes with aberrant branching (Fig. 2g,i,j) and clubby endfeet structures (Fig. 2h,k; asterisk). Further, BBS10 knockdown also led to the loss of apical enrichment of β-catenin, and significantly increased percentage of RG cells without apical endfeet (Fig. 2d,l,m). In sharp contrast to the effects of the above three genes, BBS7 knockdown caused the ventricular epithelium to invaginate towards the pial surface, resulting in rosette-like structures with actively dividing progenitors localized in the centre of the rosette lumen and migrating neurons radiating outwards (Fig. 2n,o; Supplementary Fig. 1). Taken together, these results indicate that different ciliopathy-specific genes (*BBS1*, *BBS7*, *BBS10* and *TMEM216*) modulate distinct aspects of apical–basal polarity of the RG scaffold and the integrity of the proliferative niche organization.

**Figure 3:**
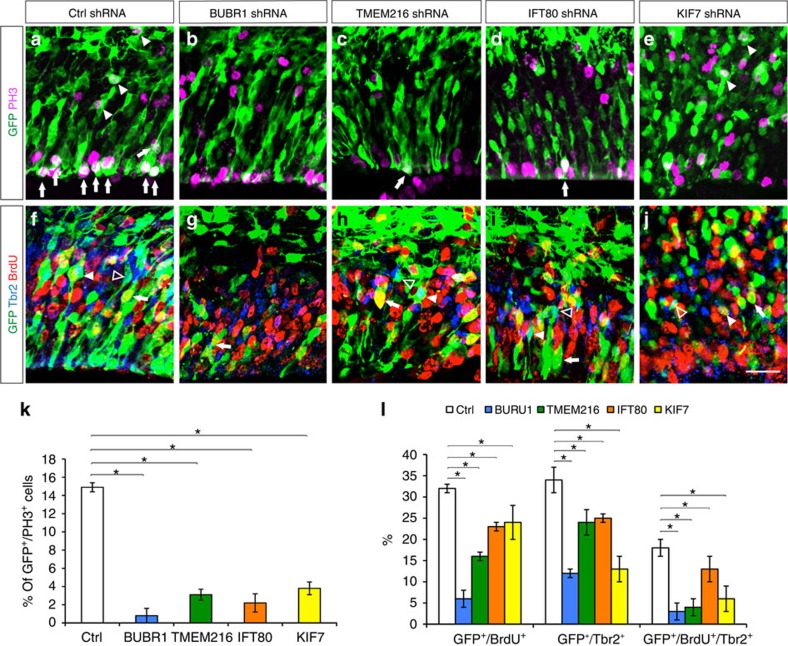
Effect of ciliopathy genes on progenitor proliferation. (**a**–**e**) Co-labelling with anti-GFP and PH3 antibodies show proliferating, GFP^+^/PH3^+^ RG progenitors in VZ (arrow) and IPs in SVZ (arrowhead), respectively, in control cortices (**a**). (**b**–**e**) In contrast, knockdown of BUBR1 [BUB1B] (**b**), TMEM216 (**c**), IFT80 (**d**) and KIF7 (**e**) resulted in significantly reduced proliferation of RG progenitors and IPs. (**f**–**j**) Co-labelling with anti-GFP, BrdU and Tbr2 antibodies indicates that knockdown of BUBR1 [BUB1B] (**g**), TMEM216 (**h**), IFT80 (**i**) and KIF7 (**j**) resulted in reduced percentage of Tbr2^+^ cells (Tbr2^+^/GFP^+^ (open arrowhead)) and mitotic progenitors (BrdU^+^/GFP^+^ (arrow), Tbr2^+^/BrdU^+^/GFP^+^ (filled arrowhead)). (**k**) Quantification of percentage of PH3^+^/GFP^+^ cells in control and BUBR1 [BUB1B], TMEM216, IFT80 and KIF7 groups. Data shown are mean±s.e.m. *P<0.05 (Student’s *t*-test). (**l**) Quantification of percentage of BrdU^+^/GFP^+^, Tbr2^+^/GFP^+^ and Tbr2^+^/BrdU^+^/GFP^+^ cells in control and BUBR1 [BUB1B], TMEM216, IFT80 and KIF7 shRNA groups. Data shown are mean±s.e.m. *P<0.05 (Student’s *t*-test). Number of brains per group=4. Scale bar, 10 μm (**a**–**j**).

As cerebral cortex forms, precise control of radial progenitor cell cycle dynamics is required for appropriate precursor pool expansion and neurogenesis. Symmetrical divisions of RG produce daughter radial progenitors and help expand the progenitor pool. As neurogenesis begins, radial progenitors divide asymmetrically and produce daughter neurons and intermediate precursor (IP) cells. IPs then undergo symmetric neurogenic divisions and predominantly give rise to the neurons of upper-layer identities. Knockdown of BUBR1 (Bub1-related kinase; BUB1B), IFT80 (intraflagellar transport 80 homologue), KIF7 (kinesin family member 7) and TMEM216 led to decreased cell divisions of both apical radial progenitors (Fig. 3a–e, arrows; Supplementary Fig. 1) and basal IPs as indicated by the reduced GFP^+^/PH3^+^ cells (Fig. 3a–e, arrowheads) and basally localized GFP^+^/Tbr2^+^, or GFP^+^/Tbr2^+^/BrdU^+^ cells (Fig. 3f–j; Supplementary Fig. 1; Supplementary Table 6). These findings suggest that distinct ciliopathy genes may selectively modulate progenitor cell division and thus, cortical neurogenesis.

### Ciliopathy genes and neuronal migration

**Figure 4:**
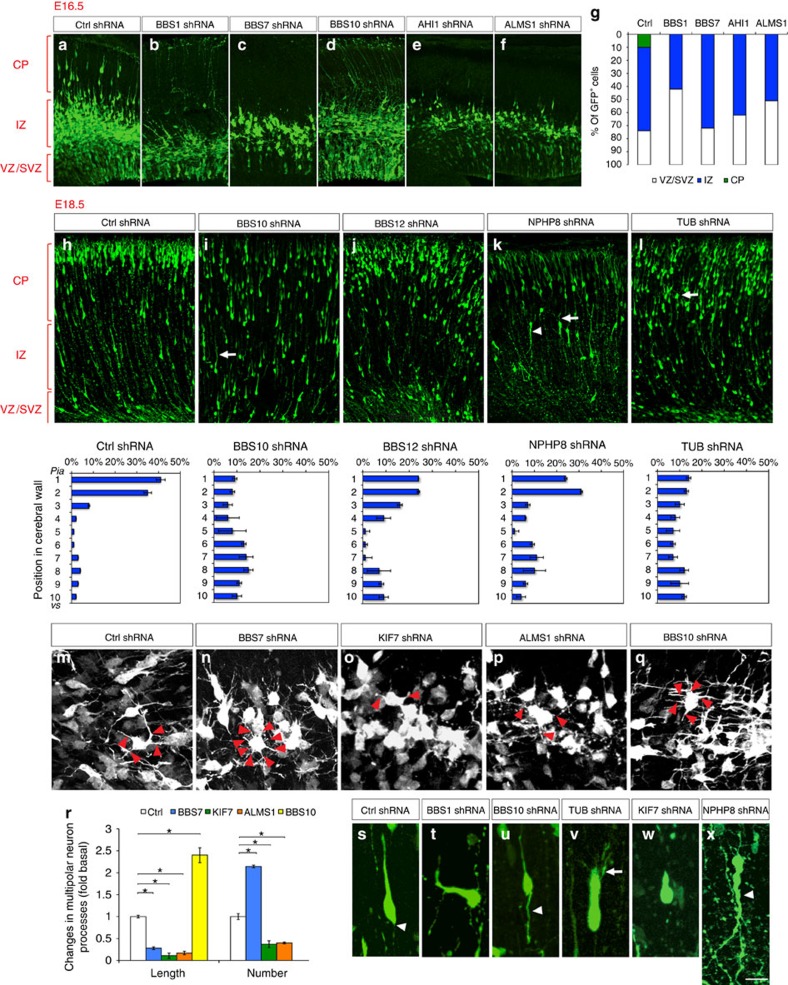
Effect of ciliopathy genes on neuronal migration. (**a**–**l**) GFP^+^ neurons express shRNAs against BBS1 (**b**), BBS7 (**c**), BBS10 (**d**,**ji**)i, AHI1 (**e**), ALMS1 (**f**), RTTN (**i**), BBS12 (**j**) and NPHP8 [RPGRIP1L] (**k**) and TUB (**l**) shRNAs do not migrate normally. shRNAs were electroporated at E14.5. **a**–**f** and **h**–**l** are from cortical analyses at E16.5 and E18.5, respectively. Arrows in **i**, **k** and **l** indicate branched leading processes. Arrowhead in **k** indicates a misoriented leading process. (**g**) Quantification of GFP^+^ cell position in the developing cortical wall (E16.5). Quantification of GFP^+^ cell position in the developing cerebral wall (E18.5) are shown under each panel in **h**–**l**. (**m**–**q**) Higher-magnification images of GFP^+^ multipolar neurons in control, BBS7, KIF7, ALMS1 and BBS10 shRNA groups. Red arrowheads indicate processes. (**r**) Quantification of average number and length of process in GFP^+^ multipolar neurons in control and BBS7, KIF7, ALMS1 and BBS10 shRNA groups. Data shown are mean±s.e.m. *P<0.05 (Student’s *t*-test). (**s**–**x**) Representative images of GFP^+^ migrating neurons from control and BBS1, BBS10, TUB, KIF7 and NPHP8 [RPGRIP1L] shRNA groups. Arrowhead, trailing process of migrating neurons. Number of brains per group=4. CP, cortical plate; IZ, intermediate zone; SVZ, subventricular zone; VZ, ventricular zone. Scale bar, 60 μm (**a**–**f**); 65 μm (**h**–**l**); 15 μm (**m**–**x**).

Newborn neurons migrate radially through the intermediate zone (IZ) to reach their specific cortical layers in the developing CP. During the initial phase of radial migration, newborn neurons within the lower IZ transiently assume a characteristic ‘multipolar’ morphology to explore their microenvironment for directional cues, and then transform into a bipolar morphology as they migrate radially (Fig. 4a). The bipolar neurons subsequently migrate towards CP by adhering to the basal RG process, with their leading process oriented towards the pial surface (Fig. 4a). Knockdown of BBS1, BBS7, BBS10, AHI1 and ALMS1 resulted in a significantly higher percentage of newborn GFP^+^ neurons remaining as Tuj-1^+^ (and Tbr2^−^) multipolar neurons in the lower IZ at E16.5 (Fig. 4b–g), suggesting a defect in the appropriate multipolar-to-bipolar transition. Notably, knockdown of these genes only delayed but not permanently stalled this transition, as indicated by the presence of bipolar migratory neurons at E18.5 in these cortices (Fig. 4; Supplementary Fig. 1). Compared with control, knockdown of BBS7, ALMS1 (Alstrom syndrome 1) and KIF7 resulted in thinner and shorter processes of the multipolar neurons, whereas knockdown of BBS10 resulted in longer processes (Fig. 4m–r; Supplementary Fig. 2; Supplementary Table 7). BBS7 knockdown also led to an increased number of processes, whereas knockdown of ALMS1 and KIF7 led to decreased number of processes in multipolar neurons (Fig. 4m–p,r; Supplementary Fig. 2; Supplementary Table 7).

In total, knockdown of 17 ciliopathy genes including *AHI1*, *ALMS1*, *BBS1*, *BBS4*, *BBS7*, *BBS9*, *BBS10*, *BBS11* [TRIM32], *BBS12*, *BUBR1* [BUB1B], *IFT80*, *KIF7*, *NPHP1* (nephronophthisis 1), *NPHP8* [RPGRIP1L], *TCTN2*, *TMEM216* and *TUB* (Tubby protein homologue) resulted in delayed migration (Fig. 4a–l; Supplementary Figs 1 and 2; Supplementary Table 7). The migration delay is associated with increased leading process branching in BBS1-, BBS4-, BBS10-, BUBR1 [BUB1B]-, IFT80-, NPHP8 [RPGRIP1L]- and TUB-deficient neurons (Fig. 4i,k,l,v (arrows); Supplementary Fig. 2d,e,h,i (arrows)), and misorientation in BBS1-, NPHP8 [RPGRIP1L]- and TCTN2-deficient neurons (Fig. 4k,t,x; Supplementary Fig. 2l (arrowhead); Supplementary Table 7). Moreover, knockdown of BBS4, BBS12, BUBR1 [BUB1B], IFT80, KIF7, NPHP1, NPHP8 [RPGRIP1L] and TUB resulted in shortened leading processes (Fig. 4; Supplementary Fig. 2; Supplementary Table 7), whereas knockdown of BBS10 and NPHP8 [RPGRIP1L] led to aberrantly elongated trailing processes (Fig. 4u,x (arrowheads)). Taken together, these results demonstrate that ciliopathy genes can regulate distinct aspects of cortical neuron migration, including the transient multipolar stage, multipolar-to-bipolar transition and glial-guided radial migration. Although control shRNA expression consistently did not reveal an altered array of migration patterns, a recent study on doublecortin and neuronal migration highlights the importance of further validation of neuronal migration effects observed with shRNAs using additional *in vivo* mouse genetic tools^14^.

### Ciliopathy genes and the identity and organization of neurons

Upon reaching the CP, newly generated neurons migrate past older neurons to occupy more superficial regions of the CP, where they terminate their migration and acquire distinct laminar and neuronal subtype identities. Compared with control, knockdown of AHI1, ALMS1, BBS1, BBS4, BBS7, BBS9, BBS10, BBS11 [TRIM32], BBS12, BUBR1 [BUB1B], IFT80, KIF7, NPHP1, NPHP8 [RPGRIP1L], TCTN2, TMEM216 and TUB resulted in a significantly increased percentage of GFP^+^/Cux1^+^ neurons localized at deeper CP positions where Ctip2^+^ or Tbr1^+^ neurons are normally localized (Supplementary Table 8; Supplementary Fig. 3). Therefore, consistent with their role in neuronal migration, knockdown of these genes impair the laminar placement of neurons with upper-layer identities. Quantification of the percentage of GFP^+^/layer marker^+^ (Cux1, Ctip2 or Tbr1) neurons indicate no significant differences between control and shRNA groups, suggesting that neuronal layer identity was not altered by shRNA expression (Supplementary Table 8). Together these data demonstrate a critical role for groups of ciliopathy genes including *AHI1*, *ALMS1*, *BBS1*, *BBS4*, *BBS7*, *BBS9*, *BBS10*, *BBS11* [TRIM32], *BBS12*, *BUBR1* [BUB1B], *IFT80*, *KIF7*, *NPHP1*, *NPHP8* [RPGRIP1L], *TCTN2*, *TMEM216* and *TUB* in the coordination of migration and placement of neurons in the developing cerebral cortex.

### Ciliopathy genes in post-migratory differentiation of neurons

**Figure 5:**
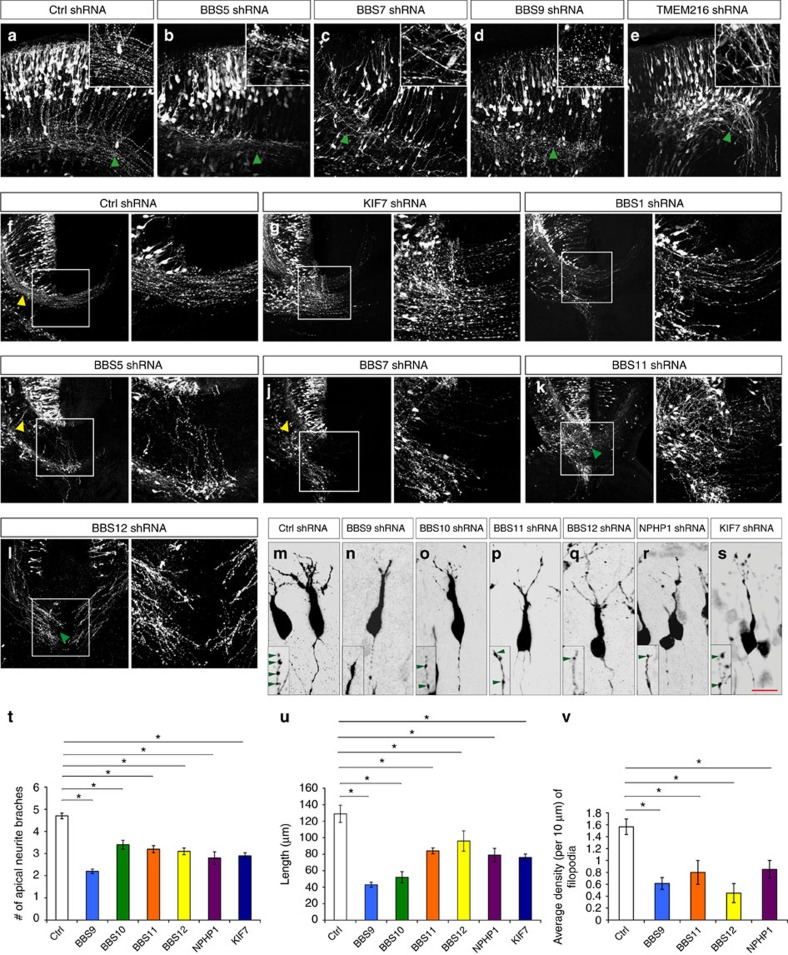
Effect of ciliopathy genes on post-migratory neuronal differentiation. Embryos were electroporated with shRNAs at E14.5 and analysed at E18.5. (**a**–**e**,**k**,**l**) GFP^+^ neurons expressing BBS5 (**b**), BBS7 (**c**), BBS9 (**d**), TMEM216 (**e**), BBS11 (**k**) and BBS12 (**l**) shRNAs display axonal fasciculation defects (compare areas indicated by green arrowheads, insets (**a**–**e**)). (**f**–**l**) GFP^+^ neurons expressing shRNA against KIF7 (**g**), BBS1 (**h**), BBS5 (**i**), BBS7 (**j**), BBS11 (**k**) and BBS12 (**l**) display midline crossing defects in the developing cerebral wall. GFP^+^ neurons expressing shRNA against BBS5 (**i**), and BBS7 (**j**) also display reduced axonal outgrowth (compare areas indicated by yellow arrowheads in **f**, **i** and **j**. Right panels in **f**–**l** show higher-magnification images of the outlined (box) area in **f**–**l**. (**m**–**s**) Neurons expressing BBS9 (**n**), BBS10 (**o**), BBS11 (**p**), BBS12 (**q**), NPHP1 (**r**) and KIF7 (**s**) shRNAs display significantly reduced total non-axonal neurite length and branching. (**t**,**u**) Quantification of average apical neurite number (**t**) and total length (**u**) in control and BBS9-, BBS10-, BBS11-, BBS12-, NPHP1- and KIF7-deficient neurons. (**v**) Quantification of filopodial density in control and BBS9-, BBS11-, BBS12- and NPHP1-deficient neurons. Data shown are mean±s.e.m. *P<0.05 (Student’s *t*-test). Number of brains per group=4. Scale bar, 80 μm (**a**–**e**); 148 μm (**f**–**l**); 18 μm (**m**–**s**).

After arriving at their laminar destination within the developing CP, projection neurons extend axons and dendrites as they assemble into functional neural circuits. During this process, neurons initially extend an axon, through the corpus callosum to the contralateral cortex or through the internal capsule to sub-cerebral targets. In addition, neurons also extend apical dendrites towards the pial surface and extensive basal dendrites from the cell soma. The dendrites of cortical neurons also develop small protrusions called spines, which are the major sites of synaptic contacts. We found that knockdown of BBS5, BBS7 resulted in significantly reduced axon outgrowth (Supplementary Table 9; Fig. 5a,f,i,j). The axons of BBS5-, BBS7-, BBS9-, BBS11 [TRIM32]-, BBS12- and TMEM216-deficient neurons also display aberrant trajectory and fasciculation (Fig. 5a–e,k,l). Knockdown of BBS1, BBS5, BBS7, BBS11 [TRIM32], BBS12 and KIF7 caused a substantial reduction in axons that project across the midline to the contralateral cortex (Fig. 5f–l; Supplementary Fig. 1; Supplementary Table 9). This defect in midline crossing in BBS5, BBS7-deficient axons is caused not only by reduced axon outgrowth but also by disrupted axon guidance. These axons reached midline, but instead of crossing, project aberrantly towards subcortical targets (Fig. 5i,j). Although the dendrites and dendritic spines are not fully formed at E18, deficiency in BBS9, BBS10, BBS11 [TRIM32], BBS12, NPHP1 or KIF7 resulted in reduced apical neurite branching and total length (Fig. 5m–u; Supplementary Fig. 1; Supplementary Table 9). In addition, BBS9, BBS11, BBS12 and NPHP1 knockdown also resulted in a lower density of filopodia (Supplementary Table 9), many of which eventually form dendritic spines^15^. In summary, these results suggest that ciliopathy-related genes *BBS1*, *BBS5*, *BBS7*, *BBS9*, *BBS10*, *BBS11* [TRIM32], *BBS12*, *KIF7*, *NPHP1* and TMEM216, differentially modulate appropriate patterns of post-migratory neuronal differentiation in cerebral cortex, including axonal growth, axonal guidance, neurite extension and arborization.

## Discussion

**Figure 6:**
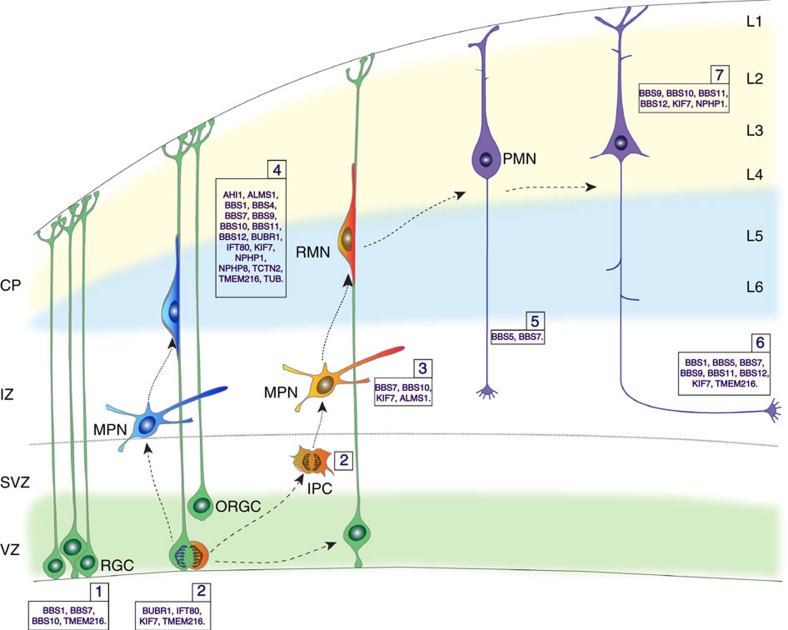
Ciliopathy genes and cerebral cortical development. Schematic of major steps (1–7) of cerebral cortical development. Polarized radial progenitor formation and organization (1), radial glial progenitor and intermediate progenitor proliferation (2), multipolar-to-bipolar transition of newborn neurons (3), radial glial-guided neuronal migration (4), axon outgrowth (5), axon guidance and fasciculation (6) and apical dendritic outgrowth (7) are illustrated. Ciliopathy-related genes identified to play a role in each of these steps are indicated. CP, cortical plate; IPC, intermediate progenitor cell; IZ, intermediate zone; L1–L6, layers 1–6; MPN, multipolar neuron; ORGC, outer radial glial cell; PMN, post-migratory neuron; RGC, radial glial cell; RMN, radially migrating neuron; SVZ, subventricular zone; VZ, ventricular zone.

Structural and functional neurological deficits are a common feature of ciliopathies in humans. However, the role of primary cilia-related genes linked to ciliopathies in brain development and function remains largely unknown. In this study, we carried out a systematic *in vivo* characterization of the role of 30 ciliopathy genes in major steps of cerebral cortical formation, ranging from progenitor development, neuronal migration, neuronal differentiation and early neuronal connectivity. Importantly, this electroporation-based approach enables the exploration of cilia-related gene functions in cerebral cortical development while avoiding hydrocephalus, noted often in cilia mutants. Our observations provide a comprehensive survey of ciliopathy gene functions in the context of cerebral cortical development. The developmental expression patterns of ciliopathy genes often correlate with their distinct functions during cerebral cortical development^16^,^17^,^18^ (Supplementary Fig. 4). Our results link deficiency in 17 of the ciliopathy genes to a variety of neurodevelopmental defects that may help us understand the diverse clinical features of ciliopathies, including cortical hypoplasia, ectopias and axonal fibre tract defects, and the resultant functional outcomes such as intellectual disabilities^1^,^4^,^19^. Currently, the known ciliopathy-related gene mutations are thought to lead to loss of function of respective proteins. The loss-of-function approach used in our screen is consistent with this, but is limited in its ability to model gain-of-function mutations or human alleles that are hypomorphic. However, further examination of these gene functions with shRNA-resistant gene constructs (Supplementary Fig. 1), gene-specific small hairpin microRNA (shmiRNA), mouse genetic tools and related human gene mutations will be necessary to fully understand the role of these genes in brain developmental abnormalities in ciliopathies^14^. Use of human mutant alleles will be particularly helpful in uncovering potential gain-of-function mutations. Gene manipulations at developmental stages other than what were described in this study may help confirm or reveal additional stage-specific effects of ciliopathy genes, especially for the ones without any detectable phenotypes in our assays. Cell biological exploration of these gene functions in cilia compartments and the associated centrosome is also necessary. Nevertheless, this study provides a template for further exploration of the biological basis of the brain structural and functional deficits associated with ciliopathies. These new insights into the functions of ciliopathy genes help link human brain malformations seen in ciliopathies to disruptions in specific cortical developmental events (Fig. 6).

The four ciliopathy genes (*BUBR1* [*BUB1B*], *IFT80*, *KIF7* and *TMEM216*) we identified as modifiers of progenitor proliferation encode proteins localized to distinct ciliary compartments (Supplementary Table 1). BUBR1 [BUB1B], localized in centrosome/basal body, is a key spindle assembly checkpoint protein, whose insufficiency leads to cell cycle misregulation and ciliogenesis defects^20^. Importantly, mutations in *BUBR1* [BUB1B] cause a rare human disorder mosaic-variegated aneuploidy or premature chromatid separation syndrome, a novel ciliopathy syndrome characterized by microcephaly and mental retardation^20^,^21^. TMEM216 is a component of transition zone and is required for centrosome/basal body docking to initiate ciliogenesis^22^,^23^,^24^. *TMEM216* is a causative gene for ciliopathy syndromes joubert syndrome and related disorders (JSRD) and Meckel–Gruber syndrome (MKS), with associated mental retardation^22^,^23^,^24^. Our observation of disrupted neural progenitor proliferation in BUBR1 [BUB1B]- and TMEM216-deficient brains is consistent with the clinical symptoms, such as microcephaly, found in human patients. On the other hand, IFT80 and KIF7 are members of IFT complexes and deficiency of IFT80 or KIF7 results in impaired ciliary signalling transduction without loss of cilia^25^,^26^,^27^. Mutations of *IFT80* cause Jeune asphyxiating thoracic dystrophy and short-rib polydactyly type III (SRP type III)^25^. Mutations in *KIF7* cause hydrolethalus and acrocallosal syndromes^26^. The four ciliopathy genes we identified as regulators of progenitor proliferation could represent two distinct molecular pathways: centrosome dependent and ciliary signalling dependent, underlying some of the cortical deficits (for example, microcephaly) associated with these gene mutations.

Knockdown of BBS1, BBS10, BBS7 and TMEM216 lead to disruption of distinct aspects of the polarized RG scaffold. BBS1, BBS10 and TMEM216 are required for the maintenance of the RG scaffold; BBS10 is required for the polarized organization of the RG scaffold, whereas knockdown of BBS7 resulted in the formation of rosette-like heterotopias caused by invagination of the progenitor niche. These observations suggest their converging yet differential influence of cilia-related genes on the RG polarity. Further understanding of the functions of these proteins localized to different ciliary compartments in the regulation of distinct aspects of RG polarity could help dissect the molecular mechanisms underlying RG organization and function. Cortical heterotopias are an abnormal brain patterning defect commonly seen in Bardet–Biedl syndrome (BBS), Joubert syndrome (JBTS), Meckel–Gruber syndrome (MKS)^9^,^13^,^28^, caused by defects in the formation and maintenance of polarized RG progenitors and RG-guided neuronal migration. Importantly, mutations in *BBS1*, *BBS7* and *BBS10* cause BBS, whereas mutations in *TMEM216* cause JBTS and MKS^19^,^24^,^29^. The presence of cortical heterotopia in these ciliopathies is associated with epilepsy and mental retardation^2^,^4^,^7^,^30^,^31^. Taken together, these data demonstrate the converging role of multiple ciliopathy genes in the development and function of RG progenitors. Disruption of these processes may underlie cortical malformations such as microcephaly and heterotopias seen in ciliopathies related to mutations in these genes.

Defects in neuronal migration and differentiation of cortical neurons can impair the organization and activity of the cortical circuitry, and are thought to underlie a broad spectrum of neurodevelopmental and psychiatric disorders^32^,^33^. Neuronal migration defects such as cortical periventricular heterotopia and polymicrogyria are common features of various ciliopathies (Supplementary Table 1). Surprisingly, our results show that 17 different ciliopathy genes modulate distinct phases of cortical neuron migration, highlighting the critical nature of primary cilia in neuronal migration during cortical development. These results also provide molecular bases and genotype–phenotype correlations for the brain abnormalities caused by aberrant neuronal migration in ciliopathies. For example, ciliopathy patients carrying mutations in *AHI1* or *BBS* genes show cortical heterotopia and polymicrogyria^29^,^34^. Consistently, our results suggest that neurons expressing AHI1 or BBS (BBS1, BBS4, BBS5, BBS7, BBS9, BBS10, BBS11 [TRIM32] and BBS12) shRNA show migration defects. We also observed that knockdown of ciliopathy genes *ALMS1*, *BUBR1* [BUB1B], *IFT80*, *NPHP1*, *NPHP8* [RPGRIP1L], *TCTN2*, *TMEM216* and *TUB* retarded neuronal migration. Neurons deficient in these genes display excessive branching or misorientated leading processes. Since knockdown of BBS1, BBS10 and TMEM216 also led to disrupted RG morphology, the migration defects seen in these three ciliopathy gene knockdowns may in part result from a compromised RG scaffold, whereas the migration defects with the other genes could be due to their specific neuronal functions. Knockdown of five genes (*BBS7*, *BBS10*, *ALMS1*, *KIF7* and *AHI1*) led to defects in the mutipolar–bipolar transition stage, suggesting a selective requirement for primary cilia in the early stages of neuronal migration. Though the molecular mechanisms involved in the transition from multipolar to radial migration are yet to be fully understood, multiple cues such as adhesion molecules (for example, TAG1, N-cadherin and Connexin 43), cytoskeletal regulators (for example, CDK5 and ARX), intracellular signalling molecules (for example, Rap1, RhoA, Mark2 and LKB1), transcription factors (for example, FoxG1, Scratch 1, 2 and KLF4) and extracellular signals such as Neurog2, Semaphorin 3A, Netrin and Reelin are involved in this process^35^,^36^,^37^,^38^,^39^,^40^,^41^,^42^,^43^. Our results suggest that primary cilium and its associated signalling proteins could help sense, integrate and convey signalling cues necessary for this transition.

Consistent with the migration defects, we found an aberrant laminar placement of neurons following disruption of 17 ciliopathy genes (*AHI1*, *ALMS1*, *BBS1*, *BBS4*, *BBS7*, *BBS9*, *BBS10*, *BBS11* [TRIM32], *BBS12*, *BUBR1* [BUB1B], *IFT80*, *KIF7*, *NPHP1*, *NPHP8* [RPGRIP1L], *TCTN2*, *TMEM216* and *TUB*). Recent studies also showed that genetic deficiency of BBS1 or BBS4 in mice results in perturbed migration and lamination defects^32^,^44^. The identification of multiple ciliopathy genes with previously unknown roles in neuronal migration and placement suggest that this stage of cortical development is highly vulnerable in ciliopathies, and the resultant changes in the formation of cortical circuits may underlie cognitive deficits associated with ciliopathies.

Post-migratory cortical neurons undergo extensive neurite outgrowth to generate the appropriate axon–dendritic architecture of the neuronal circuits. A steady and polarized delivery of membrane proteins, such as guidance cue receptors, and cytoskeletal components to the growing neurites is required to enable the neurites to extend in appropriate directional patterns as they contact and form synapses with appropriate partners. Consistently, primary cilia-mediated signalling cascades (for example, GPCR and Wnt signalling) and cilia-associated centrosome mechanisms have both been shown to regulate neuronal dendritic and axonal outgrowth and refinement^45^,^46^,^47^,^48^. We found that two BBS genes (*BBS5* and *BBS7*) are required for axonal outgrowth, whereas one transition zone gene (*NPHP1*), four BBS genes (*BBS9*, *BBS10*, *BBS11* [*TRIM32*] and *BBS12*) and an IFT regulator, *KIF7*, modulate neurite outgrowth and filopodial formation, indicating the differential and specific influence of ciliopathy genes over post-migratory neuronal differentiation.

Defects in axonal tract development, including callosal agenesis, disrupted pyramidal decussation and superior cerebellar peduncle decussation, are often seen in ciliopathy patients, and these axonal tract defects are thought to underlie some of the motor and cognitive deficits in ciliopathies^2^,^4^,^18^,^26^,^30^,^49^. It is known that midline glial structures (for example, glial wedge) and secreted guidance molecules (for example, WNT, Slit2, Shh and Netrin1) released from these structures are crucial for guiding axonal midline crossing and corpus callosum formation^49^,^50^,^51^,^52^,^53^. For example, Shh functions to guide commissural axons by acting directly as a chemoattractant for the axon growth cones. Consistently, mutations in *KIF7*, known to disrupt the Shh pathway, cause acallosal syndromes in humans and lead to dysgenesis or agenesis of the corpus callosum in mice^26^,^27^,^54^. Our observations indicated that in addition to KIF7, knockdown of five of the BBS genes, *BBS1*, *BBS5*, *BBS7*, *BBS11* [TRIM32] and *BBS12*, also led to axonal guidance and midline crossing defects. How these cilia-related proteins, localized primarily in cilium near the cell soma, modulate dynamics of the axonal growth cone remains unclear. The answer might lie in the non-ciliary expression of ciliopathy genes at other sub-cellular compartments, such as the growth cone. To date, several ciliopathy genes have been shown to have non-ciliary expression (for example, *KIF7* in the growth cone, *KIF3A*, *BBS4* and *BBS8* in synapse) or cilia-independent functions (for example, *KIF7* in axonal guidance and BBS4 in synaptic transmission), suggesting additional key roles for some of the ciliopathy genes in protein trafficking and membrane transport^54^,^55^,^56^,^57^,^58^,^59^. Future effort aimed at dissecting the role of ciliary versus non-ciliary function of ciliopathy genes during corticogenesis will be essential to delineate how they contribute to the aetiology of constellation of symptoms associated with ciliopathies.

Many of the causative genes of ciliopathy syndromes display genetic and/or direct physical interactions, leading possibly to perturbations in common cellular processes^6^,^60^. For example, it has been shown that *KIF7* genetically interacts with *BBS* loci^26^. Deficiency of *KIF7* and several *BBS* genes led to similar defects in progenitor proliferation and neuronal migration, indicating convergent biological influences of genetic interaction between *KIF7* and *BBS* genes. On the contrary, some ciliopathy-related genes can exert effects on multiple aspects of human brain development (Supplementary Table 10). For example, mutations in *AH1* can disrupt neurogenesis, neuronal migration and axon growth, leading to hypoplasia, heterotopia and failed axonal decussation in humans (Supplementary Table 10). If and how *AH1* convergently interacts with other ciliopathy genes to modulate these diverse cellular processes remains unclear.

On the basis of localization in cilia and functional interactions, ciliopathy genes have also been classified into functional networks^6^,^61^. BBSome is the best studied of the ciliopathy protein complexes. BBSome contains seven highly conserved BBS proteins (BBS1, BBS2, BBS4, BBS5, BBS7, BBS8 [TTC8] and BBS9) that localize to the basal body and functions as a unit that mediates vesicle trafficking to the ciliary membrane^62^. Three chaperonin-like BBS proteins (BBS6 [MKKS], BBS10 and BBS12) form a higher-order complex, mediate BBSome subunit association and are required for BBSome assembly^62^,^63^. Deficiency of BBS genes cause Bardet–Biedl syndrome, one of the well-characterized ciliopathies^29^. Our results show that deficiency in eight BBS protein family members (BBS1, BBS4, BBS5, BBS7, BBS9, BBS10, BBS11 [TRIM32] and BBS12) led to significant cortical defects including a disrupted RG scaffold, perturbed neuronal migration and impaired neuronal differentiation, thus highlighting the essential roles of *BBS* genes in brain development. Importantly, the results of our functional assays further clarify some of the known effects of BBSome ciliopathy in humans: it has been suggested that deficiency in any member of BBSome or chaperonin-like BBS complex disrupts the functionality of the BBSome and causes similar phenotypes in human patients and BBS animal models^60^,^63^,^64^,^65^,^66^,^67^. Consistent with this, our results show that deficiency of BBSome members and chaperonin-like BBS proteins often led to defects in common neurodevelopmental events. We found that BBS1, BBS7 and BBS10 are involved in RG progenitor development; knockdown of BBS1, BBS4, BBS7, BBS9, BBS10, BBS11 [TRIM32] or BBS12 resulted in neuronal migration defects, whereas loss of BBS1, BBS5, BBS7, BBS9, BBS10, BBS11 or BBS12 led to post-migratory, neuronal differentiation defects. However, our analysis also revealed other previously unknown distinctive requirements for individual BBSome members and chaperonin-like BBS proteins during corticogenesis. For example, knockdown of BBS1 and BBS10 resulted in excessive branching, and reduced branching of RG basal endfeet, respectively (Fig. 2). Also, knockdown of BBS7 and BBS10 led to shorter, and longer multipolar neuronal processes, respectively (Fig. 4). Thus, it is likely that even though BBSome and chaperonin-like BBS proteins function to synergize and converge on common developmental pathways, they still exert different regulatory roles during corticogenesis. The same also appears to be the case for other ciliopathy networks such as the NPHP–JBTS–MKS complex.

Ciliopathies display a broad range of clinical features, high degree of pathological variability, genetic heterogeneity and phenotypic overlaps, thus complicating their diagnosis and treatment^4^. Elucidation of multifaceted functions of cilia and associated protein complexes is fundamental to our effort to understand the biological bases of ciliopathies and develop efficient therapeutic interventions. This comprehensive *in vivo* functional investigation of the causative genes of ciliopathies in the context of cortical development not only delineates varied functions of cilia in the construction of the cerebral cortex, but also reveals potential developmental bases of brain abnormalities in ciliopathies. In the future, the use of causative human mutant alleles with defined links to cortical abnormalities in this type of screen or analysis of genotyped neural cells generated from ciliopathy patient-derived induced pluripotent stem cells can help further define the biological basis of ciliopathy mutations and enhance our understanding of cilia-related gene functions in brain development. Establishing a causal link between genotype and phenotype of ciliopathies with such approaches will be vital to develop rational therapeutic interventions for ciliopathies.

## Methods

### Mice

Mice were cared for according to guidelines approved by the University of North Carolina. Light/dark cycle in the vivarium is 7/7 h. Animals were housed in groups of three adults per cage. C57/BL6 pregnant female mice were used for *in utero* electroporation. The day of vaginal plug detection was considered E0.5.

### shRNA library of ciliopathy genes

A library of shRNAs specific to the 30 human ciliopathy genes was assembled using the resources available at the UNC Gene Therapy Center. Lists of shRNA constructs and sources are provided in the Supplementary Information (Supplementary Table 4). Plasmids used for *in utero* electroporation were prepared using the EndoFree Plasmid kit (Qiagen). For each gene, two pools of shRNAs containing two to three individual shRNAs were randomly combined and used for electroporation. Non-silencing scrambled shRNA (sc-108080, Santa Cruz), GIPZ or pLKO.1 shRNA vectors (GE Dharmacon and UNC Gene Therapy Center) were used as control. KE of shRNAs (fold change compared with control) was measured using quantitative real-time PCR and are indicated for each shRNA group. KE of 70% was used as a threshold for shRNA pools. Each pool consisted of an equivalent amount of different shRNAs (2 or 3 per pool) listed. For BBS8, only the effective pool of ShRNA containing three different shRNAs was used. shRNAs from Santa Cruz are prepooled (four shRNAs per gene) by the vendor.

### Tissue culture, transfection, RNA isolation and purification

Mouse kidney inner medullary collecting duct (IMCD3) epithelial cells were grown in DMEM/F12 media supplemented with 10% fetal bovine serum and 1% penicillin–streptomycin (P/S) antibiotics at 37 °C with 5% CO_2_. The cells were allowed to reach 80% confluency and ciliogenesis was induced by serum starvation of the cells for 24 h. IMCD3 cells were then transfected using Invitrogen Lipofectamine 2000 reagent according to the manufacturer’s instructions (Sigma). For each well of cells in a six-well plate, 1.5 μg of shRNAs and pCIG2 was used. The cells were imaged 3 days post transfection to measure transfection efficiency (percentage of GFP^+^ cells). Transfected IMCD3 cells were then lysed for RNA purification using a Qiagen RNeasy mini kit according to the manufacturer’s instructions.

### RT–PCR and qPCR

RT–PCR was performed using the Invitrogen SuperScript III First-Strand Synthesis System. Real-time PCR was performed using Applied Biosystem Power SYBR Green PCR master mix and the appropriate forward and reverse primers (Supplementary Table 5). The real-time PCR reactions were performed in triplicates. Peptidylprolyl isomerase A (Ppia) was used as an endogenous reference. The comparative (ΔΔ*C*_T_) method of relative quantification was used to determine the level of gene knockdown.

### *In utero* electroporation

*In utero* electroporations were performed as described previously^68^,^69^. Briefly, 1–2 μl of a pool of plasmid DNA (1.5 μg μl^−1^) were injected into the lateral ventricles of E14.5 brains and electroporated using five pulses at 30 V for 50 ms at 950-ms intervals through the uterine wall using a BTX ElectroSquarePorator (ECM 830). In shRNA+cDNA rescue experiments, human cDNAs were used at 1 μg μl^−1^ concentration. Each pool of shRNAs or control vectors was electroporated into four different embryos. Embryos were then allowed to develop for 2 or 4 days prior to analyses.

### Immunohistochemistry

The primary antibodies used were anti-RC2 (1:1, Iowa Hybridoma), anti-β-catenin (rabbit polyclonal, 1:1,000;), anti-GFP (chicken, 1:1,000; Abcam), anti-BrdU (mouse monoclonal, 1:50; BD Biosciences), anti-phospho-histone H3 (PH3) (rabbit polyclonal, 1:200; Millipore), anti-Tbr2 (1:500, AB23345, Abcam), anti-Ctip2 (1:500, AB18645, Abcam), anti-Cux-1 (1:100, SC13024, Santa Cruz Biotechnology), anti-Tbr1 (1:500, AB31940, Abcam) and anti-Brn1 (1:1,000, gift from A. Ryan, McGill University)^10^. Secondary antibodies were AlexaFluor 488 or Cy3-conjugated (Invitrogen and Jackson ImmunoResearch). Nuclei were counterstained with 4',6-diamidino-2-phenylindole (Sigma). Electroporated mouse brains were removed and fixed with 4% paraformaldehyde overnight at 4 °C. Brains were then embedded in 3% agarose and sectioned at 50 μm with a Vibratome (VT1000S; Leica Microsystems). Sections were blocked with PBS/10% goat serum/0.2% Triton X-100 for 1 h, and incubated in primary antibodies (see above) overnight at 4 °C. After three washes in 1 × PBS, sections were incubated with secondary antibodies (1:1,000) at room temperature for 2 h, washed and mounted with Citifluor anti-fading solution (Agar). For BrdU labelling, BrdU (50 mg kg^−1^) was injected intraperitoneally into pregnant mice 1 h prior to brain tissue processing.

### Quantitative analysis of progenitor proliferation, polarity and niche organization

GFP^+^ RG progenitors were co-immunolabelled with RC2 or anti-nestin antibodies. The characteristic apical endfeet attached to the pial surface and branched basal endfeet were imaged and quantified per section in control and shRNA brains. Lack of apical endfeet and increased or decreased branching of basal endfeet were measured. The apical molecular polarity of RG was detected by apical β-catenin enrichment at the ventricular surface of GFP^+^ radial progenitors. Changes in RG progenitor proliferation were detected using PH3 and BrdU immunolabelling. The number and position of GFP^+^/BrdU^+^ or PH3^+^ nuclei in the ventricular zone was measured (25 × 10^3^ μm^2^ area) to examine changes in the rate of cell division and the position of progenitor nuclei during cell cycle. Actively proliferating intermediate progenitors in control and shRNA brains were co-labelled with anti-Tbr2 and anti-BrdU antibodies and quantified (25 × 10^3^ μm^2^ area) to monitor the changes in their proliferation patterns.

### Quantitative analysis of neuronal migration and placement

Cerebral wall was divided into 10 equal bins and the percentage of GFP^+^ neurons in each bin was counted^68^. Number of leading processes per neuron was counted in migrating neurons in the IZ. The percentage of migrating neurons with leading processes oriented at an angle <75° to the pial surface was also measured. Percentage of GFP^+^/layer marker^+^(Tbr1, Ctip2 or Cux1) neurons located below CP, away from their normal laminar locations, was quantified to detect changes in the laminar placement of shRNA-expressing neurons. Images were acquired using Zeiss 710 and Zeiss 780 confocal systems. Imaging analysis was done using Zeiss LSM Image Browser and ImageJ software (NIH).

### Quantitative analysis of post-migratory differentiation of neurons

Number of GFP^+^, shRNA-expressing neurons in the CP displaying an axon and the projection of the axon apically and then laterally towards contralateral cortical or subcortical regions was quantified per section. The length of apical and basal neurites and the number of the primary neurite branches were counted per GFP^+^ neuron. The density of immature filopodia on primary apical neurites was quantified per 10-μm length.

### Statistical analysis

GraphPad or Excel was used for data analysis. Two-tailed Student’s *t*-test and two-way analysis of variance with Tukey–Kramer multiple comparison test were performed using GraphPad. Data were collected and processed blindly. All data are expressed as means±s.e.m. Statistical comparisons between different knockdown groups were not performed, as there are differences in the efficacy of the knockdown of different genes.

## Additional information

**How to cite this article:** Guo, J. *et al.* Developmental disruptions underlying brain abnormalities in ciliopathies. *Nat. Commun.* 6:7857 doi: 10.1038/ncomms8857 (2015).

## Supplementary Material

Supplementary Information Supplementary Figures 1-4, Supplementary Tables 1-10 and Supplementary References
