# Supplementary material for: Developmental disruptions underlying brain abnormalities in ciliopathies
Source: Nat Commun. 2015 Jul 24;6:7857. doi: 10.1038/ncomms8857 (PMC4515781; doi:10.1038/ncomms8857)
Supplement: Supplementary Information — Supplementary Figures 1-4, Supplementary Tables 1-10 and Supplementary References [file ncomms8857-s1.pdf]

Supplementary Information

Supplemental Figures

Figure S1

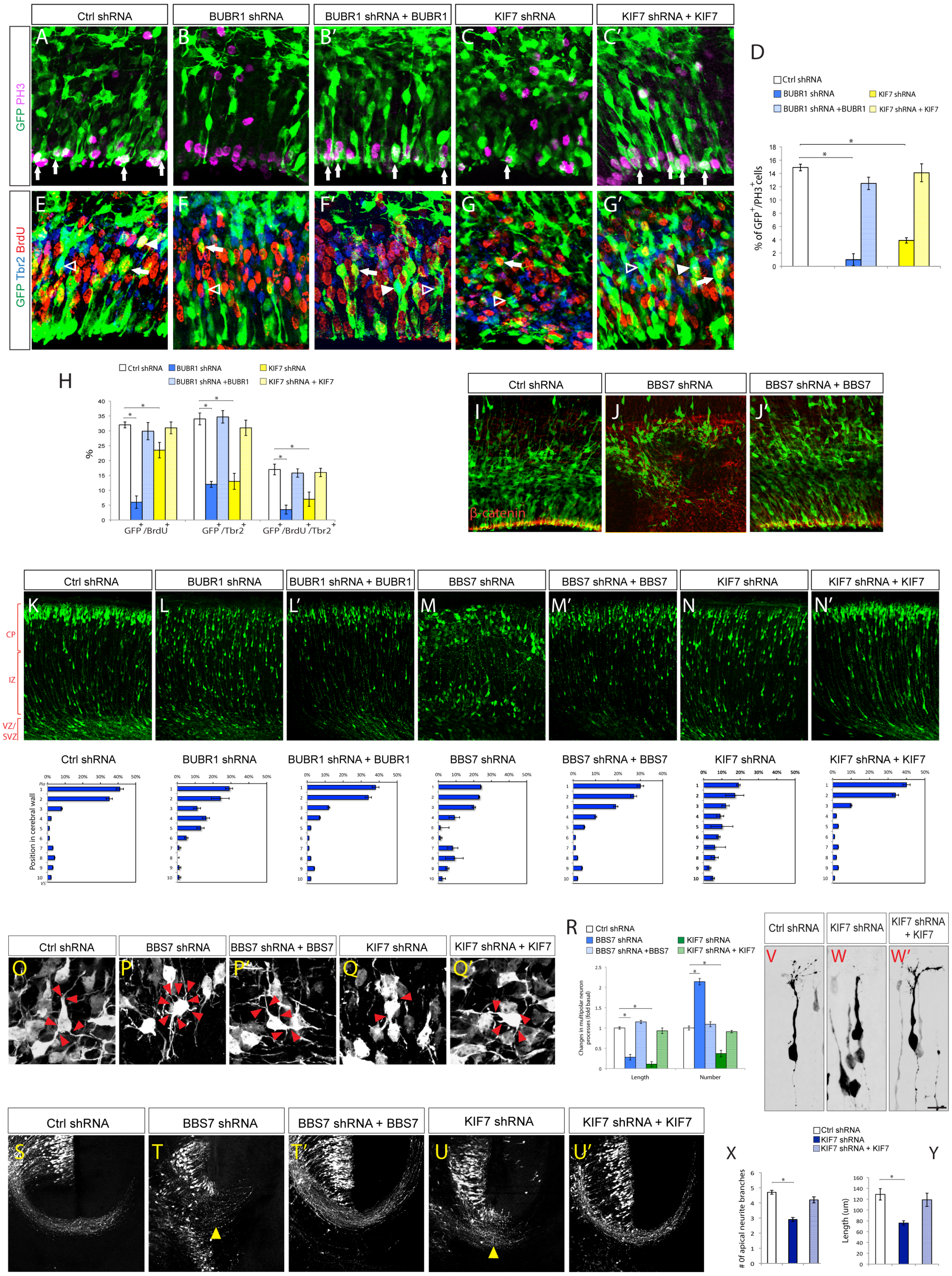

Figure S1. Rescue of BBS7, BUBR1 [BUB1B], and KIF7 shRNA mediated effects in the developing cerebral cortex. BBS7, BUBR1 [BUB1B], and KIF7 shRNAs disrupted distinct aspects of progenitor development, neuronal migration, and axon growth. Expression of human BBS7, BUBR1, or KIF7 (which are not targeted by murine shRNAs) rescued the respective shRNA phenotypes. (A-D) Proliferating GFP^+/$PH3^+$ RG progenitors in VZ (arrow) in control cortices (A). (B-C) In contrast, knockdown of BUBR1 (B) and KIF7 (C) resulted in significantly reduced proliferation of RG progenitors (D). Expression of human BBS7 or KIF7 rescued the shRNA phenotypes (B', C', D). (E-H) Co-labeling with anti-GFP, BrdU and Tbr2 antibodies indicates that knockdown of BUBR1 (F) and KIF7 (G) resulted in reduced percentage of $Tbr2^+$ cells ($Tbr2^+$/GFP^+ [open arrowhead]) and mitotic progenitors ($BrdU^+$/GFP^+[arrow], $Tbr2^+$/$BrdU^+$/GFP^+[filled arrowhead]; H). (F', G', H) Expression of human BUBR1 or KIF7 rescued these defects. (I, J, J') BBS7 shRNA disrupted VZ organization (J), which was rescued with BBS7 (J'). (K-N) GFP^+ neurons express shRNAs against BUBR1 (L), BBS7 (M), and KIF7 (N) do not migrate normally. shRNAs were electroporated at E14.5 and cortices were analyzed at E18.5. (L', M', N') Rescue of migration defects with BUBR1, BBS7 and KIF7. (K''-M'') Quantification of GFP^+ cell position in the developing cortical wall. (O-R). Altered multipolar neuronal branching in BBS7 (P) and KIF7 (Q) shRNA expressing cortices, rescued with human BBS7 (P') and KIF7 (Q'), respectively (R). Red arrowheads (O-Q) indicate processes. (S-U) GFP^+ neurons expressing shRNA against BBS7 (T) and KIF7 (U), display midline crossing defects (arrowhead, T, U) in the developing cerebral wall. (T', U') Rescue of crossing defects with human KIF7 and BBS7, respectively. (V-Z) Neurons expressing KIF7 (W) shRNAs display significantly reduced total non axonal-neurite length and branching. (X') KIF7 rescue of shRNA effect. Quantification of average apical neurite number (Y) and total length (Z). Data shown are mean ± SEM. $^*P<0.05$ (Student's *t*-test). Number of brains/ group= 4. Scale bar: A-G, 12.5μm; I-J, 42μm; K-N, 67μm; O-Q, 9μm; S-U, 125μm; V-W, 15μm. CP, cortical plate; IZ, intermediate zone; SVZ, subventricular zone; VZ, ventricular zone.

Figure S2

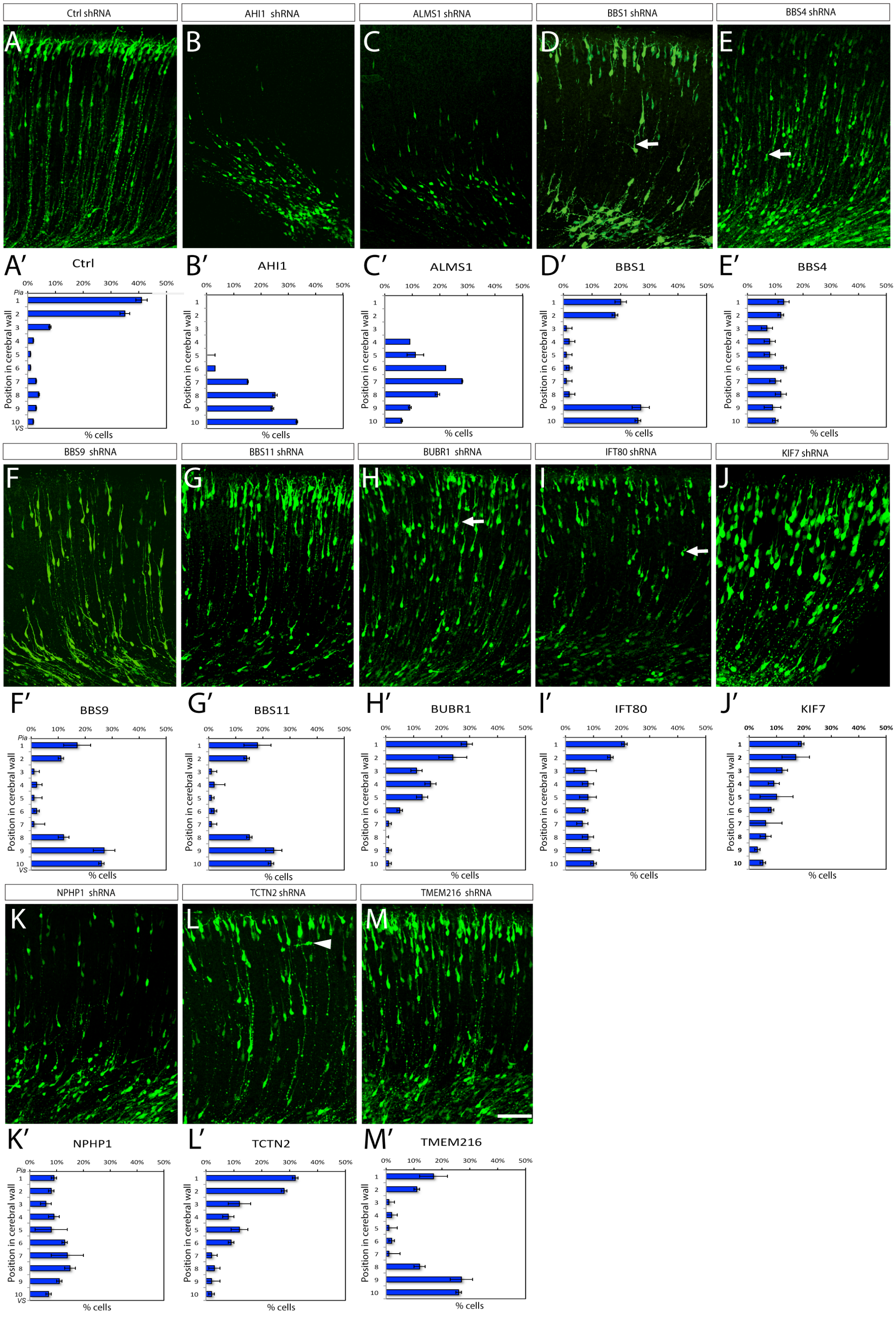

Ctrl shRNA
AHI1 shRNA
ALMS1 shRNA
BBS1 shRNA
BBS4 shRNA
A
B
C
D
E
A′ Ctrl
B′ AHI1
C′ ALMS1
D′ BBS1
E′ BBS4
0% 10% 20% 30% 40% 50%
Pia
VS
Position in cerebral wall
% cells
BBS9 shRNA
BBS11 shRNA
BUBR1 shRNA
IFT80 shRNA
KIF7 shRNA
F
G
H
I
J
F′ BBS9
G′ BBS11
H′ BUBR1
I′ IFT80
J′ KIF7
NPHP1 shRNA
TCTN2 shRNA
TMEM216 shRNA
K
L
M
K′ NPHP1
L′ TCTN2
M′ TMEM216

Figure S2. Ciliopathy gene deficiency and neuronal migration. (A-M) E14.5 embryonic cortices were electroporated and the position of GFP^+ neurons in the development cerebral cortex were analyzed at E18.5. Representative images are shown (also see Table 5). AHI1, ALMS1, BBS1, BBS4, BBS9 [TRIM32], BBS11, BUBR1 [BUB1B], IFT80, KIF7, NPHP1, TCTN2 and TMEM216 shRNAs significantly retarded neuronal migration in different ways as compared to control shRNA. Arrows [D, E, H, I] indicate branched leading processes. Arrowhead [L] indicates misoriented leading process. (A'-M') Quantification of GFP^+ cell position in the developing cortical wall (E18.5). Number of brains/ group= 4. Scale bar, A-C, E, G-I, K, M, 80μm; D, J, F, L, 68μm.

Figure S3

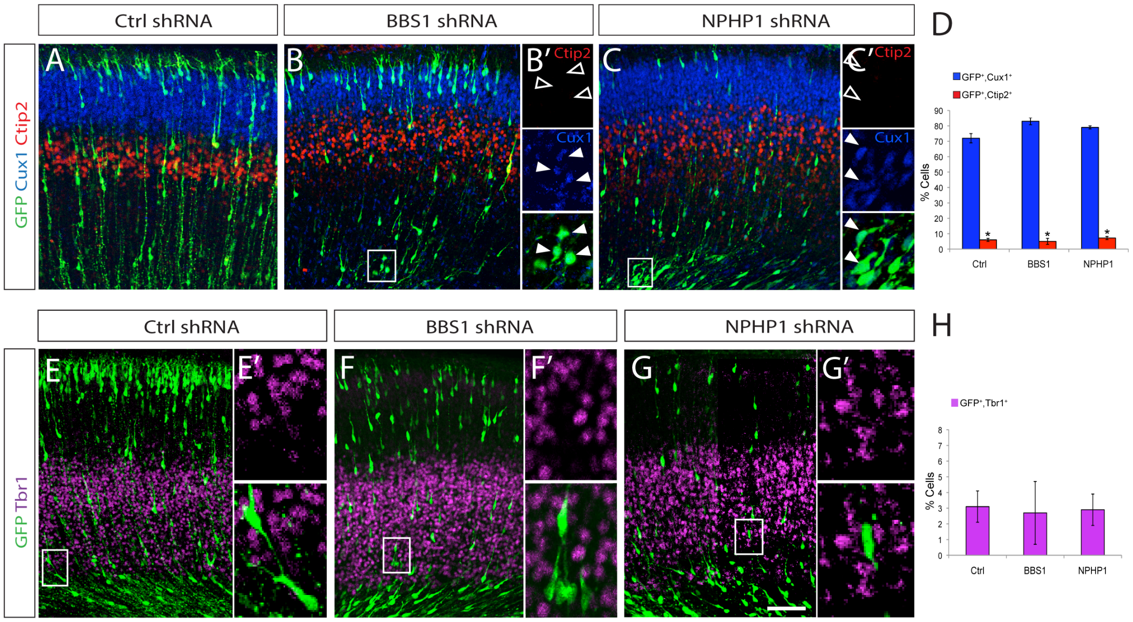

Figure S3. Effect of ciliopathy genes on neuronal identity. (A-G) GFP^+ neurons were co-labeled with antibodies to Cux1 (layer II/III; A-C), Ctip2 (layer V; A-C), and Tbr1 (layer VI; E-G). Even though aberrantly migrating BBS1 (B) or NPHP1 (C) shRNA expressing neurons expressed Cux1 and were ectopically localized away from the emerging layers, the percentage shRNA expressing (GFP^+) neurons that co-labeled with Cux1 ($GFP^+/Cux1^+$), Ctip2 ($GFP^+/Ctip2^+$), or Tbr1($GFP^+/Tbr1^+$) was not altered compared to control (D, H). (B', C') Higher magnification images of outlined areas in (B)

and (C) show ectopically localized neurons are Cux1(arrowhead), but not Ctip2 (open arrowheads) positive. (E'-G') Higher magnification images of outlined areas in E-G show GFP^+ neurons are not Tbr1 positive. (D, H) Quantification of the percentage of GFP^+/layer $marker^+$ neurons. No significant changes were observed, indicating shRNA expression affected migration and placement but not neuronal identity. Data shown are mean ± SEM. $^*P \geq 0.05$ (Student's *t*-test). Number of brains/ group= 4. Scale bar: A-C, 90μm; E-G, 85μm.

Figure S4

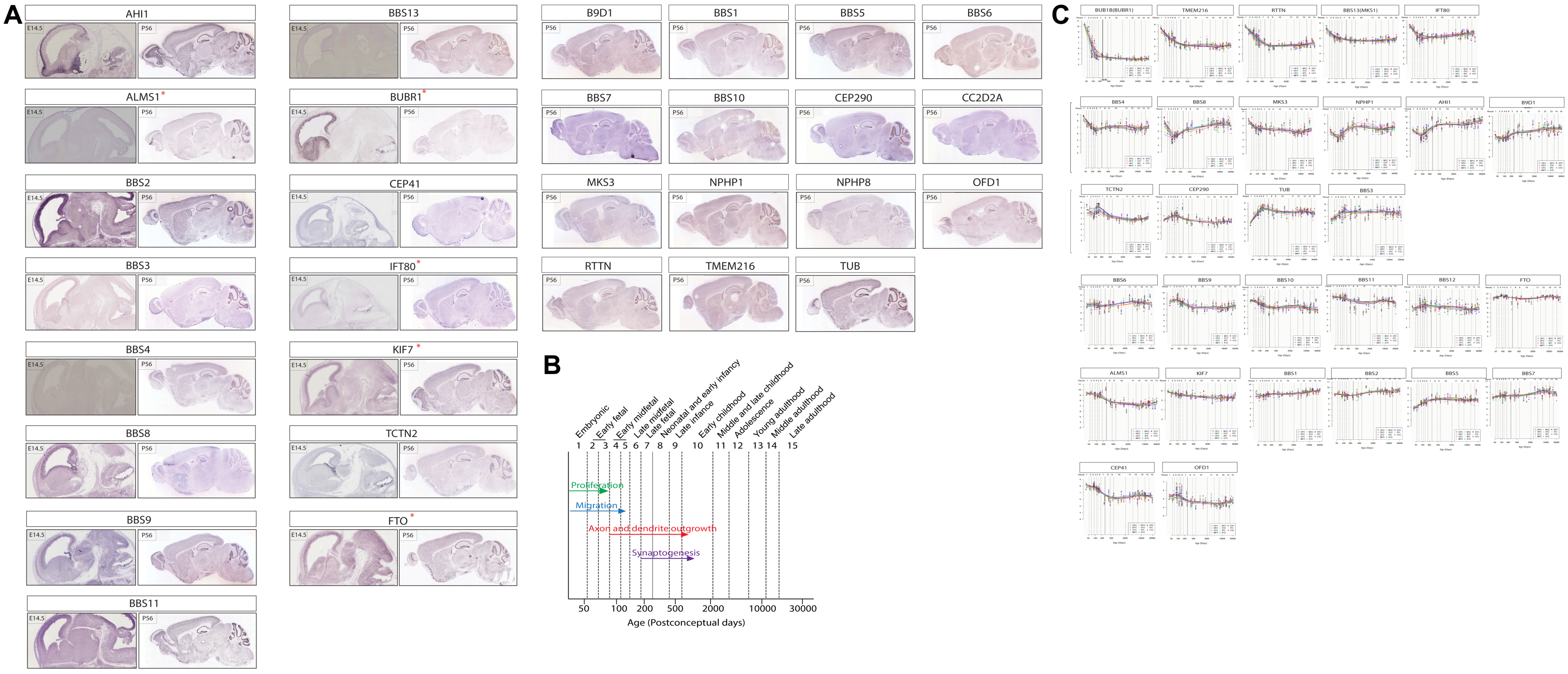

A
AHI1
ALMS1*
BBS2
BBS3
BBS4
BBS8
BBS9
BBS11
BBS13
BUBR1*
CEP41
IFT80*
KIF7*
TCTN2
FTO*
B9D1
BBS1
BBS5
BBS6
BBS7
BBS10
CEP290
CC2D2A
MKS3
NPHP1
NPHP8
OFD1
RTTN
TMEM216
TUB
E14.5
P56
B
1 Embryonic
2 Early fetal
3
4 Early midfetal
5
6 Late midfetal
7 Late fetal
8 Neonatal and early infancy
9 Late infance
10 Early childhood
11 Middle and late childhood
12 Adolescence
13 Young adulthood
14 Middle adulthood
15 Late adulthood
Proliferation
Migration
Axon and dendrite outgrowth
Synaptogenesis
50
100
200
500
2000
10000
30000
Age (Postconceptual days)
C
BUB1B(BUBR1)
TMEM216
RTTN
BBS13(MKS1)
IFT80
BBS4
BBS8
MKS3
NPHP1
AHI1
B9D1
TCTN2
CEP290
TUB
BBS3
BBS6
BBS9
BBS10
BBS11
BBS12
FTO
ALMS1
KIF7
BBS1
BBS2
BBS5
BBS7
CEP41
OFD1

Figure S4. Developmental expression of ciliopathy genes. The expression patterns of the tested ciliopathy genes in mouse cerebral cortex [E14.5 (http://www.eurexpress.org/ee/)[98] and P56 (http://www.brain-map.org)][99] and human neocortex (http://hbatlas.org)[100] (B-C). (A) In mouse, BUBR1, IFT80, and KIF7 show enriched expression in the proliferative zones at E14.5 (A, red asterisk), consistent with our observation that BUBR1, IFT80 and KIF7 are regulators of progenitor proliferation. In humans, the expression of BUBR1, TMEM216 and IFT80 gradually declined from early embryonic stages till perinatal stage, consistent with their role in progenitor proliferation[100,101,102]. Thus, both mouse and human expression data support our results indicating that BUBR1, TMEM216, IFT80, and KIF7 are regulators of cortical progenitor development. In human neocortex, the expression of TCTN2 and TUB show gradual increase from early to mid fetal stages, corresponding to the time window during which neuronal migration occurs[100,101,102]. This pattern TCTN2 and TUB expression is consistent with their detected role as regulators of neuronal migration. Further, human transcriptome trajectories suggest that the majority of the BBS genes, BBS1, BBS2, BBS3 [ARL6], BBS5, BBS6 [MKKS], BBS7, BBS9, BBS10, BBS11 [BUB1B], and BBS12 have widespread and relatively stable expression pattern during neocortical development, consistent with our results indicating that these BBS protein members play multiple roles during cerebral cortical formation.

Supplemental Tables

Table S1. shRNA library of ciliopathy genes

Gene	Protein family	Protein complex	Ciliary localization	Ciliary function	Associated ciliopathy	Brain expression	Brain abnormalities in humans
AHI1	ND	ND	Basal body/Centrosome[1]	Ciliogenesis, ciliary-dependent WNT signaling[2]	JBTS	Yes	Cerebellar vermis hypoplasia, deepened interpeduncular fossa, elongated superior cerebellar peduncles cortical heterotopias, polymicrogyria[3,4]
CEP41	Polyglutamylase enzyme	ND	Basal body/Centrosome[5]	Glutamylation in the ciliary axoneme[5]	JBTS	Yes	
BBS1	BBS	BBSome[6,7]	Basal body/centrosome, cilium[6,7]	Ciliogenesis[6,7], ciliary GPCR trafficking[8,9]	BBS	Yes	Frontal cortical dysplasia, microcephaly, cortical and cerebellar atrophy, cortical heterotopias, polymicrogyria[10]
BBS2	BBS				BBS, MKS	Yes	
BBS4	BBS				BBS, MKS	Yes	
BBS5	BBS				BBS	Yes	
BBS7	BBS				BBS	Yes	
TTC8 (BBS8)	BBS				BBS	Yes	
BBS9	BBS				BBS	Yes	
ARL6 (BBS3)	BBS, small GTPase	ND	Basal body/centrosome[11]	Ciliary localization of BBSome[11], ciliary GPCR trafficking[8]	BBS	Yes	
MKKS (BBS6)	type II chaperonin superfamily	type II chaperonin	Basal body/centrosome[12]	BBSome assembly[12]	BBS, MKS, MKKS	Yes	
BBS10					BBS	Yes	
BBS12					BBS	Yes	
TRIM32 (BBS11)	E3 Uniquitin ligase	ND	Basal body/centrosome[13], cytoplasma	ND	BBS	Yes	
MKS1 (BBS13)	Tripartite motif protein	NPHP-JBTS-MKS, Transition	Transition zone[14-16], basal body, Cilium (TMEM216,	Ciliogenesis centrosome and ciliary membrane	BBS, MKS	Yes	Cerebellar vermis hypoplasia, deepened interpeduncular fossa,

TMEM67 (MKS3)	Tetraspan transmembrane protein	zone complex[14-16]	CEP290)	composition[14-18], ciliary signal transduction[18]	BBS, MKS, JBTS	Yes	elongated superior cerebellar peduncles, cortical heterotopias, polymicrogyria[3,4,17,19-22]
TMEM216	Tetraspan transmembrane protein				JBTS, MKS	Yes	
CEP290 (BBS14)	BBS				BBS, MKS, JBTS, NPH, LCA, SLS	Yes	
CC2D2A (MKS6)	BBS				MKS, JBTS	Yes	
TCTN2	Tectonic family				MKS, JBTS	Yes	
NPHP1	Nephrocystin family				JBTS, NPH, SLS	Yes	
RPGRIP1L (NPHP8)	RPGRIP1 family				MKS, JBTS	Yes	
B9D1	B9D family				MKS	Yes	
ALMS1	ND	ND	Basal body/Centrosome[23,24]	Ciliogenesis, cilium-dependent PCP signaling[23,24]	ALST	Yes	Enlarged ventricle, gray and white matter atrophy, brain lesions[25]
BUB1B (BUBR1)	BUB family	ND	Basal body/Centrosome[26]	Ciliogenesis[26]	PCS[27]	Yes	Microcephaly, brain hypoplasia, corpus callosal agenesis, enlarged forth ventricle and posterior fossa, cerebellar vermis hypoplasia, hydrocephalus[28]
IFT80	IFT	IFT-B	Cilium	Shh signal transduction[29]	ATD[29]	Yes	Cerebellar vermis hypoplasia, deepened interpeduncular fossa, elongated superior cerebellar peduncles[3,30]
KIF7	Kinesin family	IFT-A	Basal body, cilium	Shh signal transduction[31,32]	HLS2, ACLS JBST	Yes	Hydrocephalus, corpus callosal agenesis[31]
OFD1	ND	ND	Basal body/Centrosome[33]	Ciliogenesis[33]	OFD	Yes	Intracerebral cysts, corpus callosal agenesis, cerebellar agenesis, porencephaly, pachygyria and heterotopias, hydrocephalus, cerebral or cerebellar atrophy, and berry aneurysms[34,35]
TUB	Tubby family	ND	Basal body/Centrosome	Ciliary GPCR trafficking[8,9,36,37]	Obesity	Yes	Brain atrophy[38]

FTO	2-oxoglutarate-dependent nucleic acid demethylases	ND	ND	Cilia maintanence and ciliary dependent WNT signaling[39]	Obesity	Yes	

AHI1, Abelson helper integration site 1; ALMS1, Alstrom syndrome 1; ARL6, ADP-ribosylation factor-like 6; B9D1, B9 protein domain 1; BBS, Bardet–Biedl syndrome; BUB1B, BUB1 mitotic dheckpoint serine/threonine kinase B; BUBR1, Bub1-related protein; CC2D2A, coiled-coil and C2 domain containing 2A; CEP 41, Centrosomal protein 41; FTO, fat mass and obesity associated; IFT80, intraflagellar transport 80 homolog (Chlamydomonas); KIF7, Kinesin family member 7; MKKS, McKusick-Kaufman syndrome; MKS, Meckle Syndrome; NPHP, Nephronophthisis; OFD1, Oral-facial-digital syndrome 1; RPGRIP, retinitis pigmentosa GTPase regulator interacting protein; TMEM, transmembrane protein; TRIM32, tripartite motif containing 32; TTC8, tetratricopeptide repeat domain 8; TUB, tubby bipartite transcription factor; ACLS, Acrocallosal syndrome; ALST, Alstrom syndrome; ARPKD, autosomal recessive polycystic kidney disease; JATD, Jeune asphyxiating thoracic dystrophy; HLS2, Hydrolethalus syndrome 2; JBTS, Joubert syndrome; LCA, Leber congenital amaurosis; MKS, Meckel-gruber syndrome; MKKS, McKusick-Kauffman syndrome; NPH, nephronophthisis; PCS, Premature chromatid separation; SLS, Senior-Loken Syndrome; ND, not defined.

Table S2. Other ciliopathy genes

Gene	Protein family	Protein complex	Ciliary localization	Ciliary function	Associated ciliopathy	Brain expression	Brain abnormalities in humans
ARL13B	Arf like small GTPases	ND	Cilium	Cilia maintanence, ciliary signal transduction[40]	JBTS	Yes	Cerebellar vermis hypoplasia, deepened interpeduncular fossa, elongated superior cerebellar peduncles[3]
CSPP1	Centrosomal protein	ND	Basal body and axoneme	Ciliary length and ciliogenesis[41]	JBTS	Yes	
TMEM138	Tetraspan transmembrane protein	ND	Transition zone and cilium	Ciliogenesis centrosome and ciliary membrane composition[14-18], ciliary signal transduction[18,42]	JBTS	Yes	
TMEM237	Tetraspan transmembrane protein	ND	Transition zone	Ciliogenesis, WNT signaling	JBTS	Yes	
INPP5E	Lipid 5-phophotase	ND	Cilium	Cilia maintanence, ciliary signal transduction[43]	JBTS	Yes	
NPHP2	Nephrocystin family	Transition zone complex	Transition zone	Ciliogenesis centrosome and ciliary membrane composition[14-18], ciliary signal transduction[18]	NPHP, SLS	Yes	Cerebellar vermis hypoplasia, enlarged fourth ventricle[44,45]
NPHP3	Nephrocystin family	Transition zone complex	Transition zone		NPHP, MKS, SLS	Yes	
NPHP4	Nephrocystin family	Transition zone complex	Transition zone		NPHP, SLS	Yes	
NPHP5	Nephrocystin family	ND	Basal body	Ciliogenesis[46]	NPHP, LCA, SLS	Yes	
NPHP7	Nephrocystin family, Glis2 family	ND	Basal body	Ciliary signal transduction[47]	NPHP	Yes	
NPHP9	Nephrocystin family	ND	Basal body	Ciliary signal transduction[48]	NPHP	Yes	
ATXN10	Ataxin family	Transition zone complex	Transition zone	Ciliary signal transduction[45]	NPHP, spinocerebellar ataxia	Yes	
CEP83	Centrosomal protein	ND	Basal body	Ciliogenesis[49]	NPHP	Yes	Hydrocephalus[49]
C5orf42	ND	ND	ND	ND[50]	OFDVI, JBTS	Yes	Elongated superior cerebellar peduncle, thin corpus callosum, cortical atrophy[50]

SCLT1	ND	ND	Basal body	Ciliogenesis[51]	OFDIX	Yes	Microcephaly, corpus callosal agenesis, pachygyria, cerebellar vermis agenesis[51]
TBC1D32	TBC1 domain family	ND	Basal body	Ciliogenesis[51]	OFDIX	Yes	
PKD1	Polycystin family	ND	Cilium	Ciliary signal transduction[52]	PKD	Yes	Brain aneurysms[53]
PKD2	Polycystin family	ND	Cilium	Ciliary signal transduction[52]	PKD	Yes	
PKHD1	Fibrocystin	ND	Cilium	Ciliary signal transduction	PKD	Yes	
DNAHC5	Dynein protein family	Motor protein complex	Cilium	Cilia maintanence	PCD	Yes	Hydrocephalus[54]
DNAI1	Dynein protein family	Motor protein complex	Cilium	Cilia maintenance[55]	PCD	Yes	ND
CEP120	Centriolar protein	ND	Basal body	Ciliogenesis[56]	JATD	Yes	Cerebellar vermis hypoplasia, deepened interpeduncular fossa, elongated superior cerebellar peduncles, ventricle dilatation[3,30,57]
NEK1	NIMA-related kinase	ND	Basal body	Ciliogenesis, cilia maintenance	PKD, SRP[58,59]	Yes	
DYNC2H1	Dynein protein family	Motor protein complex	Cilium	Cilia maintenance	JATD, SRP[58,60]	Yes	
IFT139	IFT	IFT-B	Transition zone and cilium	Ciliogenesis, Shh signal transduction[61]	JATD, NPH	Yes	
CENPF	Centromere protein family	Centromere-kinetochore complex	Centrosome	Ciliogenesis	Microcephaly[62]	Yes	Microcephaly, cerebellar vermis hypoplasia, corpus callosal agenesis
KATNB1	Katanin family	Microtubule-severing complex	Centrosome	Ciliogenesis	Microcephaly[63]	Yes	Microcephaly, thinning of corpus callosum[63]
IFT88	IFT	IFT-B	Basal body and cilium	Ciliogenesis, Shh signal transduction, mitotic spindle orientation	Anosmic[64]	Yes	ND
AIPL1	LCA	ND	Cilium	ND	LCA[65]	No	ND
LCA5	LCA	ND	Basal body and cilium	Ciliary protein transport	LCA[65,66]	No	ND

GUCY2D	Guanylate cyclase	ND	Cilium	ND	LCA[65]	No	ND
NMNAT1	Adenylyl transferase 1	ND	ND	ND	LCA[65]	No	ND
RD3	Retinal protein	ND	Photoreceptor cells	Protein transport	LCA[65]	No	ND
RDH12	Retinol dehydrogenase	ND	Photoreceptor cells	ND	LCA[65,67]	No	ND
RPE65	Retinoid isomerohydrolas e	ND	Retinal pigment epithelial cells	ND	LCA[65]	No	ND
LRAT	Lecithin retinol acyltransferase	ND	Retinal pigment epithelial cells	ND	LCA[65]	No	ND
IMPDH1	IMP-dehydrogenase	ND	Photoreceptor cells	ND	LCA[65,68]	Yes	Absent septum pellucidum, cerebellar vermis hypoplasia[69]
CRX	Cone-rod homeobox protein	ND	Eye ciliary margin	ND	LCA[65,70]	Yes	
TULP1	Tubby like protein 1	ND	Photoreceptor cells	Photoreceptor protein transport	LCA[65,71]	Yes	
KCNJ13	Inwardly rectifying potassium channel family of protein	ND	Photoreceptor cells	ND	LCA[65,72]	Yes	
CRB1	The Crumbs family	The Crumbs complex	Basal body	Cilia maintenance	LCA[65,73]	Yes	
MERTK	Tyrosine kinase	ND	Retinal pigment epithelial cells	ND	LCA[65]	Yes	
SPATA7	LCA	ND	Retina	ND	LCA[65,74]	Yes	
RPGRIP1	GTPase regulator interacting protein	ND	Photoreceptor cells	ND	LCA[65]	Yes	
POC1B	Centriolar protein	ND	Basal body	Ciliogenesis[75]	LCA, JBTS, PKD	Yes	Cerebellar vermis hypoplasia, deepened interpeduncular fossa, elongated superior cerebellar peduncles
CCDC28B (MGC1203)	Coiled coil domain-containing protein	ND	Basal body	Ciliogenesis, ciliary length regulation and signal transduction	BBS	Yes	Frontal cortical dysplasia, microcephaly, cortical and cerebellar atrophy, cortical heterotopias[10]
IFT172	IFT	IFT-B	Transition zone and cilium	Ciliary protein transport, ciliogenesis[76]	BBS	Yes	

IFT27	IFT	IFT-B	Transition zone and cilium	Cilioary protein transport, ciliogenesis[77]	BBS	Yes	
BBIP1	ND	BBSome	Basal body	BBSome complex assembly[78]	BBS	Yes	
CYS1	Cystin	ND	Cilium	ND	ARPKD	Yes	Brain aneurysms[53]
KIF11	Kinesin family	ND	Cilium	Ciliary signal transduction[79]	Microcephaly	Yes	Microcephaly
KIF14	Kinesin family	ND	Cilium	Ciliary signal transduction[79]	Microcephaly	Yes	Microcephaly
KIF2A	Kinesin family	ND	Cilium	Ciliary signal transduction[79]	Microcephaly	Yes	Microcephaly
KIF5C	Kinesin family	ND	Cilium	Ciliary signal transduction[79]	Microcephaly	Yes	Microcephaly
EVC	Ellis van Creveld syndrome protein	EVC complex	Cilium	Ciliary signal transduction	EVC	Yes	Hypoplastic ventricle and hydrocephalus[80]
EVC2	Ellis van Creveld syndrome protein	EVC complex	Cilium	Ciliary signal transduction	EVC	Yes	
RTTN	ND	ND	Basal body[81]	Cilia maitenance[81]	PMG[81]	Yes	Polymicrogyria, lateral ventricle dialatation[81]

AIPL1, Aryl hydrocarbon receptor interacting protein-like 1; ARL13B, ADP-ribosylation factor-like protein 13B; ATXN10, ataxin10; BBIP1, BBsome interacting protein 1; C5orf42, chromosome 5 open reading frame 42; CCDC28B, Coiled coil domain- containing protein 28B; CENPF, Centromere protein F; CEP83, centrosomal protein 83kDa; CEP120, centrosomal protein 120kDa; CRB1, Crumbs family member 1; CRX, Cone-rod homeobox protein; CSPP1, centrosome and spindle pole associated protein 1; CYS1, Cystin 1; DNAHC5, dynein, axonemal, heavy chain 5; DNAI1, dynein, axonemal, intermediate chain 1; DYNC2H1, dynein, cytoplasmic 2, heavy chain 1; EVC, Ellis van Creveld syndrome; EVC2, Ellis van Creveld syndrome 2; GUCY2D, Guanylase cyclase 2D; IFT27, intraflagellar transport 27; IFT88, intraflagellar transport 88; IFT139, intraflagellar transport 139; IFT172, intraflagellar transport 172; IMPDH1, IMP (inosine 5'-monophosphate) dehydrogenase 1; INPP5E, Inositol polyphosphate-5-phosphatase; KATNB1, katanin p80 subunit B1; KCNJ13, potassium inwardly-rectifying channel, subfamily J, member 13; KIF11, Kinesin family member 11; KIF14, Kinesin family member 14; KIF2A, Kinesin family member 2A; KIF5C, Kinesin family member 5C; LCA5, Leber congenital amaurosis 5; LRAT, lecithin retinol acyltransferase (phosphatidylcholine--retinol O-acyltransferase); MERTK, c-mer proto-oncogene tyrosine kinase; NEK1, NIMA (never in mitosis gene a)-related kinase 1; NMNAT1, nicotinamide nucleotide adenylyltransferase 1; NPHP, Nephronophthisis; PKD, Polycystic kidney disease; PKHD1, Polycystic kidney and hepatic disease 1; RD3, Retinal degeneration 3; RTTN, rotatin; SCLT1, sodium channel and clathrin linker 1; SPATA7, spermatogenesis associated 7; TBC1D32, TBC1 domain family, member 32; TMEM, transmembrane protein; RPE65, Retinal pigment epithelium-specific protein 64kDa; RPGRIP1, retinitis pigmentosa GTPase regulator interacting protein 1; ARPKD, autosomal recessive polycystic kidney disease; BBS, Bardet–Biedl syndrome; JATD, Jeune asphyxiating thoracic dystrophy; JBTS, Joubert syndrome; LCA, Leber congenital amaurosis; MKS, Meckel-gruber syndrome; MKKS, McKusick-Kauffman syndrome; NPH, nephronophthisis; PCD, Primary ciliary dyskinesia; PKD, Polycystic kidney disorder; PMD, Polymicrogyria; SLS, Senior-Loken Syndrome; SRP, Short-rib-polydactyly syndrome; ND, not defined.

Table S3. Human MRI phenotypes associated with ciliopathy gene mutations

Gene	Human MRI phenotypes
AHI1	Molar tooth sign (MTS), cortical polymicrogyria, cerebellar vermis hypoplasia, corpus callosal agenesis[4]
ALMS1	Enlarged ventricle, gray and white matter atrophy, brain lesions[25]
ARL13B	Molar tooth sign (MTS), cerebellar vermis hypoplasia, elongated superior cerebellar peduncle[82,97]
ATXN10	Cerebral atrophy[45]
BBS2	Cerebral ventricular dilatation, neuronal ectopias[83]
BBS4	Cerebral ventricular dilatation, neuronal ectopias, corpus callosal agenesis[83]
BBS6	Corpus callosal hypoplasia[83]
BUBR1	Microcephaly, brain hypoplasia, enlarged forth ventricle and posterior fossa, cerebellar vermis hypoplasia[26]
C5orf42	Molar tooth sign (MTS), enlarged superior cerebellum peduncle, cortical atrophy, thin corpus callosum[50]
CC2D2A	Molar tooth sign (MTS), cerebellar vermis hypoplasia, thickened superior cerebellar peduncle[22]
CEP41	Molar tooth sign (MTS), cerebellar vermis hypoplasia, thickened superior cerebellar peduncle[5]
CEP120	Molar tooth sign (MTS), hydrocephalus, cerebellar hypoplasia[56]
CEP83	Hydrocephalus[49]
CEP290	Molar tooth sign (MTS), thickened and maloriented superior cerebellar peduncles and cerebellar vermis hypoplasia[21]
CSPP1	Molar tooth sign (MTS), thickened and maloriented superior cerebellar peduncles and cerebellar vermis hypoplasia, occipital encephalocele, cortical heterotopia[41]
FTO	Brain atrophy[38]
INPP5E	Molar tooth sign (MTS), cerebellar vermis hypoplasia, elongated superior cerebellar peduncle[84]
KATNB1	Microcephaly, enlarged lateral ventricles, thinning of corpus callosum, simplified gyri[63]
KIF7	Molar tooth sign (MTS), hydrocephalus, corpus callosal agenesis, deep interpeduncular fossa and stretched cerebellar peduncles[31]
KIF14	Cerebral hypoplasia, cerebellar hypoplasia, corpus callosal agenesis, cerebellar vermis agenesis[79]
MKS3	Molar tooth sign (MTS), malformation of the posterior fossa, with severe vermis hypoplasia or vermis aplasia, global cerebellar hypoplasia associated with subtentorial cystic dilatation of the cisterna magna[85]
NPHP1	Inferior vermis hypoplasia, molar tooth sign (MTS), elongated superior cerebellar peduncle[86,87]
NPHP8	Molar tooth sign (MTS), occipital encephalocele, anencephaly[88]
OFD1	Corpus callosal agenesis, hippocampal hypoplasia, interhemispheric cysts, periventricular heterotopia, molar tooth sign (MTS), cerebellar vermis hypoplasia[35]
POC1B	Molar tooth sign (MTS), cerebellar vermis hypoplasia[75]
RTTN	Polymicrogyria[81]
SCLT1	Corpus callosal agenesis, microcephaly, inferior cerebellar vermis agenesis, pachygyria[51]
TBC1D32	Corpus callosal agenesis, microcephaly, inferior cerebellar vermis agenesis, pachygyria[51]
TCTN2	Molar tooth sign (MTS), cerebellar vermis aplasia[45]
TMEM237	Molar tooth sign (MTS), cerebellar vermis hypoplasia, elongated superior cerebellar peduncle, posterior encephalocele, abnormal shaped forth ventricle[89,90]

Functional gene modules	Ciliary localization	Human clinical manifestations^
BBSome (BBS1, 2, 4, 5, 7, 8, 9)	Axoneme	Ataxia, rod-cone dystrophy, cerebellar atrophy, corpus callosal agenesis, ventricle enlargement, intellectual disabilities [10,45,83,91]

NPHP1-4-8 module	Transition zone	Ataxia, molar tooth sign (MTS), intellectual disabilities[15,45,87,92]
NPHP5-6, ATXN10 module	Basal body/Centrosome	Ataxia, molar tooth sign (MTS), cerebellar atrophy, intellectual disabilities[45,46,88]
MKS1-6, TCTN1, 2, 3 module	Transition zone	Occipital encephalocele, molar tooth sign (MTS), intellectual disabilities[14,15,20,45]

AHI1, Abelson helper integration site 1; ALMS1, Alstrom syndrome 1; ARL13B, ADP-ribosylation factor-like protein 13B; ATXN10, Ataxin10; BBS2, Bardet-Biedl syndrome 2; BBS4, Bardet-Biedl syndrome 4; BBS6, Bardet-Biedl syndrome 6; BUBR1, Bub1-related kinase; C5orf42, chromosome 5 open reading frame 42; CC2D2A, coiled-coil and C2 domain containing 2A; CEP 41, Centrosomal protein 41; CEP83, Centrosomal protein 83; CEP120, Centrosomal protein 120; CEP 290, Centrosomal protein 290; CSPP1, centrosome and spindle pole associated protein 1; FTO, fat mass and obesity associated; INPP5E, Inositol polyphosphate-5-phosphatase; KATNB1, Katanin p80 subunit B1; KIF7, Kinesin family member 7; KIF14, Kinesin family member 14; MKS3, Meckle Syndrome 3; NPHP, Nephronophthisis; OFD1, Oral-facial-digital syndrome 1; POC1B, POC centriolar protein B; RTTN, rotatin; SCLT1, sodium channel and clathrin linker 1; TBC1D32, TBC1 domain family, member 32; TCTN2, tectonic family member 2; TMEM237, transmembrane protein 237. ^, Only the clinical manifestations common to mutations in all members of the complex are listed.

Table S4. shRNA library of ciliopathy genes

Gene Name	Accession No.	shRNA constructs	Source	Knockdown efficiency
AHI1	NM_026203.2	TRCN0000191207<br>TRCN0000202060<br>TRCN0000190390<br>TRCN0000201363<br>TRCN0000192100	GE Dharmacon	Pool1<br>Yes (78%)
				Pool 2<br>Yes (76%)
ALMS1	NM_145223.2	TRCN0000183842<br>TRCN0000183730<br>TRCN0000180565<br>TRCN0000183191	GE Dharmacon	Pool1<br>Yes (87%)
				Pool 2<br>Yes (79%)
B9D1	NM_013717.2	V3LMM_505274<br>V3LMM_505275<br>V3LMM_505276<br>V3LMM_505277	GE Dharmacon	Pool1<br>No (5%)
				Pool 2<br>No (8%)
BBS1	NM_001033128	V3LMM_449353<br>V3LMM_449358<br>V3LMM_449357<br>V3LMM_449355<br>V3LMM_449354<br>V3LMM_449356	GE Dharmacon	Pool1<br>Yes (91%)
				Pool 2<br>Yes (79%)
BBS2	NM_026116	TRCN0000177554<br>TRCN0000176447<br>TRCN0000177437<br>TRCN0000182334	UNC Gene Therapy Center	Pool1<br>Yes (75%)
				Pool 2<br>Yes (82%)
BBS3 (ARL6)	NM_019665	TRCN0000100844<br>TRCN0000100842<br>TRCN0000100841<br>TRCN0000100840<br>TRCN0000100843	UNC Gene Therapy Center	Pool1<br>Yes (76%)
				Pool 2<br>Yes (82%)
BBS4	NM_175325	V2LMM_88124<br>V2LMM_88126<br>V2LMM_227338<br>V2LMM_88128<br>V2LMM_197617	GE Dharmacon	Pool1<br>Yes (81%)
				Pool 2<br>Yes (78%)
BBS5	NM_028284	V2LMM_40947<br>V2LMM_38072<br>V3LMM_513848	GE Dharmacon	Pool1<br>Yes (87%)
				Pool 2<br>Yes (82%)
BBS6 (MKKS)	NM_021527	V2LMM_73798<br>V2LMM_66409<br>V2LMM_66373	GE Dharmacon	Pool1<br>No (16%)
				Pool 2<br>No (22%)
BBS7	NM_027810.3	V2LMM_54540<br>V3LMM_498514	GE Dharmacon	Pool1<br>Yes (76%)

		V3LMM_498516<br>V3LMM_498518<br>V3LMM_498517		Pool 2<br>Yes (72%)
BBS8 (TTC8)	NM_029553.3	TRCN0000113210<br>TRCN0000113211<br>TRCN0000113212<br>TRCN0000113213<br>TRCN0000113214	GE Dharmacon	Pool1<br>No (34%)<br>Pool 2<br>Yes (75%)
BBS9	NM_178415.1	TRCN0000182387<br>TRCN0000182069<br>TRCN0000178683<br>TRCN0000182647<br>TRCN0000181485	GE Dharmacon	Pool1<br>Yes (70%)<br>Pool 2<br>Yes (72%)
BBS10	NM_027914	V2LMM_99446<br>V2LMM_99447<br>V2LMM_99445<br>V2LMM_99444<br>V2LMM_197526	GE Dharmacon	Pool1<br>Yes (70%)<br>Pool 2<br>Yes (75%)
BBS11 (TRIM32)	NM_053084.2	TRCN0000040832<br>TRCN0000040831<br>TRCN0000040830<br>TRCN0000040829<br>TRCN0000040828	GE Dharmacon	Pool1<br>Yes (77%)<br>Pool 2<br>Yes (83%)
BBS12	NM_001008502.2	TRCN0000178910<br>TRCN0000179579<br>TRCN0000179253<br>TRCN0000183292	GE Dharmacon	Pool1<br>Yes (90%)<br>Pool 2<br>Yes (73%)
BUBR1 (BUB1B)	NM_009773.3	sc-37543-SH	Santa Cruz	Pool1<br>Yes (79%)<br>Pool 2<br>Yes (87%)
CEP41	NM_031998.2	sc-142283-SH	Santa Cruz	Pool1<br>Yes (83%)<br>Pool 2<br>Yes (87%)
CEP290	NM_146009.2	V2LMM_205703<br>V2LMM_25106<br>V2LMM_23922	GE Dharmacon	Pool1<br>No (26%)<br>Pool 2<br>No (12%)
CC2D2A	NM_172274.1	TRCN0000181757<br>TRCN0000197491<br>TRCN0000197895<br>TRCN0000178278<br>TRCN0000177905	GE Dharmacon	Pool1<br>Yes (76%)<br>Pool 2<br>Yes (89%)
FTO	NM_011936	TRCN0000183897<br>TRCN0000179651<br>TRCN0000180978<br>TRCN0000178985	GE Dharmacon	Pool1<br>Yes (78%)<br>Pool 2<br>Yes (79%)

IFT80	NM_026641.2	TRCN0000190083<br>TRCN0000193055<br>TRCN0000191159<br>TRCN0000190186<br>TRCN0000191678	GE Dharmacon	Pool1<br>Yes (71%)<br>Pool 2<br>Yes (77%)
KIF7	NM_001291222.1	TRCN0000090441<br>TRCN0000090439<br>TRCN0000116760	GE Dharmacon	Pool1<br>Yes (86%)<br>Pool2<br>Yes (81%)
MKS1	NM_017777.3	TRCN0000136854<br>TRCN0000138085<br>TRCN0000136766<br>TRCN0000137356<br>TRCN0000137209	GE Dharmacon	Pool1<br>Yes (86%)<br>Pool 2<br>Yes (73%)
MKS3	NM_177861	TRCN0000125984<br>TRCN0000125985<br>TRCN0000125986<br>TRCN0000125987<br>TRCN0000125988	GE Dharmacon	Pool1<br>Yes (73%)<br>Pool 2<br>Yes (79%)
NPHP1	NM_016902.3	TRCN0000192281<br>TRCN0000200670<br>TRCN0000201440<br>TRCN0000190911	GE Dharmacon	Pool1<br>Yes (76%)<br>Pool 2<br>Yes (82%)
NPHP8 (RPGRIP1L)	NM_173431.2	TRCN0000106076<br>TRCN0000106077<br>TRCN0000106078<br>TRCN0000106079	GE Dharmacon	Pool1<br>Yes (77%)<br>Pool 2<br>Yes (71%)
OFD1	NM_177429.3	TRCN0000191091<br>TRCN0000191237<br>TRCN0000191531<br>TRCN0000192215<br>TRCN0000191532	GE Dharmacon	Pool1<br>Yes (79%)<br>Pool 2<br>Yes (73%)
TCTN2	NM_026486.3	V2LMM_14487<br>V2LMM_12203<br>V3LMM_473359	GE Dharmacon	Pool1<br>Yes (86%)<br>Pool 2<br>Yes (74%)
TMEM216	NM_001277860.1	sc-154446-SH	Santa Cruz	Pool1<br>Yes (75%)<br>Pool 2<br>Yes (79%)
TUB	NM_021885	TRCN0000034508<br>TRCN0000034507<br>TRCN0000034506<br>TRCN0000034505<br>TRCN0000034504	GE Dharmacon	Pool1<br>Yes (79%)<br>Pool 2<br>Yes (77%)

Table S5. Quantitative realtime-PCR primers

Gene Name	Realtime-PCR primers sequences
AHI1	Forward: CCGGTGGTCCGCAAAG<br>Reverse: GGCGGCTTCTGCTTGGT
ALMS1	Forward: TCTGGTGCTTGTGATACGAAAGA<br>Reverse: AGGCCCGGAGTGAATGTG
B9D1	Forward: TGCAATGGCTGCAGCAA<br>Reverse: CCTGCCCGGTGATCATGA
BBS1	Forward: GGCTGCGGCGTCTTCA<br>Reverse: CATTTAGAATTGGCTTCGTTGCT
BBS2	Forward: TGAAACTGCGCCACAAAATC<br>Reverse: CGTCGTAGCGCCCTATGG
BBS3 (ARL6)	Forward: TTAAAGACAAGCCGTGGCATATT<br>Reverse: TCCTGCAGGCCTTCTCCTT
BBS4	Forward: ACGTGAGGCCTCGCATGTA<br>Reverse: GAGTAACGGGCTTTCATCGTTT
BBS5	Forward: ATCGCAGTGCACAGAGCATA<br>Reverse: CAGGTTCACGATCTTGTTGCTTA
BBS6 (MKKS)	Forward: GAATACACGTCTGCCCGTAAGAT<br>Reverse: AAGCTCCCGCAAAGGAAAG
BBS7	Forward: GAAGCAAGCAGAGCCTGATCA<br>Reverse: TGATTACATGTGGGACGTCCAT
BBS8 (TTC8)	Forward: GGGCACATAGCTGTGGGAAT<br>Reverse: GGGCCAGCCTGAAGCAT
BBS9	Forward: GCAGCCTCACCCCTCTTACA<br>Reverse: AAGTCCGGATGCTCACAGAGA
BBS10	Forward: CGGTTCTGGGTCTTGTTGAAC<br>Reverse: CTGCCGAAGCATCTTAAATGC
BBS11 (TRIM32)	Forward: GGACCTCTTGACGGGAATTTC<br>Reverse: CCATCCTCTGGCACATCTTCA
BBS12	Forward: TTCCTCTTGGATGGCCTCAT<br>Reverse: GCCAGGGACTTCAGCACAGT

BUBR1 (BUB1B)	Forward: GGCCATGTTGTTTGGTACCAGTA<br>Reverse: TTCGCTGTGTTGGAGAAGATTC
CEP41	Forward: CGCTCCCAACTTGGTCCTT<br>Reverse: GGTCCAGCCCGTGTAGCAT
CEP290	Forward: GATGCTGTCATGGACCAGATCA<br>Reverse: AGAAGATCCGTGAGCTCTCGAA
CC2D2A	Forward: GGTGGCACGCACCAATG<br>Reverse: ACTTACTCCCAGGCTCCTGTATCA
FTO	Forward: TCTCCTAGAATTCCCCACTCATAGA<br>Reverse: TTGGGAACTGGGTGCTTCA
IFT80	Forward: GCTCCCTGGTGCACATCAG<br>Reverse: TTGGAGCTGCTCACATACTCATG
KIF7	Forward: GCCGAGGCAGCCACATC<br>Reverse: GCATTCCCTCCCAGAGAGTCT
MKS1	Forward: CCCCGTGCGCAACCT<br>Reverse: TTGCTTGATGTGATTCTTTGCA
MKS3	Forward: CCTTAAGAGAGAGGCGGAAAATT<br>Reverse: CGTCTGCCCATCCGTGTT
NPHP1	Forward: CCGCCTCTGCCTGTTTGA<br>Reverse: ACACGGCTCGAACTGTATGGA
NPHP8 (RPGRIP1L)	Forward: GCAGATGAAAGTGCAGATTGCT<br>Reverse: GACTTCGGTTTTGTCTGTAAGATCAG
OFD1	Forward: GGGCGTGATGCCATGAA<br>Reverse: GGGATGACTGCGCTCTCATT
TCTN2	Forward: TTCCCCCGGCTTTGAAG<br>Reverse: GTAACTGGCATCCGCGAAGT
TMEM216	Forward: GTGCACGTACATCTGTCCTACATG<br>Reverse: GCCCAAGAGCCCACCATTA
TUB	Forward: GCCTCCCCTTCTCTGTATTCCT<br>Reverse: CACCTGGCATCCTGAAATCC

Table S6. Ciliopathy genes and cortical progenitor development

Gene	Radial glial Scaffold organization	Apical β-catenin enrichment	Basal endfeet morphology	Apical endfeet morphology	Changes in % of $PH3^+/GFP^+$ cells (Fold basal)	Changes in % of $BrdU^+/GFP^+$ cells (Fold basal)	Changes in % of $Tbr2^+/GFP^+$ cells (Fold basal)	Changes in % of $BrdU^+/Tbr2^+/GFP^+$ cells (Fold basal)
BBS1	Disrupted	Normal	Disrupted	Normal	•	•	•	•
BBS7	Disrupted	Normal	Normal	Normal	•	•	•	•
BBS10	Disrupted	Disrupted	Disrupted	Disrupted	•	•	•	•
BUBR1	Normal	Normal	Normal	Normal	↓ (0.07± 0.07)	↓ (0.19 ± 0.09)	↓ (0.35 ± 0.03)	↓ (0.21 ± 0.12)
IFT80	Normal	Normal	Normal	Normal	↓ (0.15 ± 0.07)	↓ (0.72 ± 0.03)	↓ (0.72 ± 0.03)	↓ (0.71± 0.18)
KIF7	Disrupted	Normal	Normal	Normal	↓ (0.26 ± 0.05)	↓ 0.73 ± 0.11)	↓ (0.38 ± 0.08)	↓ (0.41 ± 0.16)
TMEM216	Disrupted	Normal	Disrupted	Normal	↓ (0.20 ± 0.05)	↓ (0.50 ± 0.03)	↓ (0.71 ± 0.05%)	↓ (0.23 ± 0.11)

No significant changes were observed in AHI1, ALMS1, B9D1, BBS2, BBS3, BBS4, BBS5, BBS6, BBS8, BBS9, BBS11, BBS12, CEP41, CEP290, CC2D2A, FTO, MKS1, MKS3, NPHP1, NPHP8, TCTN2, OFD1, and TUB shRNA groups. Data shown are mean (± SEM) fold basal changes. Abbreviations used: •, no significant changes; ↓, decreased, p value<0.05 (Student's t test). Number of embryos/group = 4. Total number of cells analyzed: $PH3^+/GFP^+$ [Control (523), BUBR1 (678), TEME216 (586), IFT80 (632), KIF7 (602)], $Tbr2^+/BrdU^+/GFP^+$ [Control (629), BUBR1 (536), TMEM216 (637), IFT80 (579), KIF7 (646)]. Also see Figures 2 and 3. Qualitative changes in radial glial morphology were analyzed in 15 serial sections obtained from 4 different embryonic brains.

Table S7. Ciliopathy genes and neuronal migration

Gene	Changes in # of branches of multipolar migrating neurons (fold basal)	Changes in length of processes of multipolar migrating neurons (fold basal)	Changes in # of branches of bipolar migrating neurons (fold basal)	Changes in length of leading process of bipolar migrating neurons (fold basal)	Changes in leading process orientation^ of bipolar neurons (fold basal)	Altered extent of migration*
AHI1	•	•	•	•	•	↓
ALMS1	↓ (0.4 ± 0.02)	↓ (0.17 ± 0.04)	•	•	•	↓
BBS1	•	•	↑ (2.1 ± 0.24)	•	Yes (6.7 ± 1.2)	↓
BBS4	•	•	↑ (1.8 ± 0.7)	↓ (0.45 ± 0.13)	•	↓
BBS7	↑ (2.14 ± 0.03)	↓ (0.28 ± 0.07)	•	•	•	↓
BBS9	•	•	•	•	•	↓
BBS10	•	↑ (2.4 ± 0.37)	↑ (1.7 ± 0.3)	•	•	↓
BBS11	•	•	•	•	•	↓
BBS12	•	•	•	↓ (0.42 ± 0.19)	•	↓
BUBR1	•	•	↑ (1.8 ± 0.2)	↓ (0.67 ± 0.33)	•	↓
IFT80	•	•	↑ (1.6 ± 0.3)	↓ (0.29 ± 0.15)	•	↓
KIF7	↓ (0.37 ± 0.08)	↓ (0.11 ± 0.06)	•	↓ (0.17 ± 0.11)	•	↓
NPHP1	•	•	•	↓ (0.58 ± 0.24)	•	↓
NPHP8	•	•	↑ (2.2 ± 0.7)	↓ (0.51 ± 0.21)	Yes (7.3 ± 2.1)	↓
TCTN2	•	•	•	•	Yes (5.4 ± 1.7)	↓
TMEM216	•	•	•	•	•	↓
TUB	•	•	↑ (3.1 ± 0.8)	↓ (0.57 ± 0.13)	•	↓

Data shown are mean (± SEM) fold basal changes. No significant changes were observed in BBS2, BBS3, BBS5, BBS6, BBS8, MKS1, MKS3, CEP290, CC2D2A, B9D1, OFD1, FTO, and CEP41 shRNA groups. Abbreviations used: •, no significant changes; ↑, increased; ↓, decreased, p value<0.05 (Student's t test). Changes in the extent of migration were tested with two-way ANOVA. Number of embryos / group = 4. Number of cells analyzed: number and length of multipolar neuronal branches [Control (31), BBS7 (32), KIF7 (36), ALMS1 (25), BBS10 (33)], number and length of bipolar neurites [Control (60), BBS1 (76), BBS4 (62), BBS10 (72), BUBR1 (64), IFT80 (63), NPHP8 (61), TUB (60)], leading process orientation [Control (51), NPHP8 (51), TCTN2 (36)], extent of migration [Control (749), AHI1 (523), ALMS1 (437), BBS1 (605), BBS4 (645), BBS7 (703), BBS9 (499), BBS10 (721), BBS11 (742), BBS12 (743), BUBR1 (641), IFT80 (634), KIF7 (690), NPHP1 (633), NPHP8 (589), TCTN2 (625), TMEM216 (712), TUB (596)]. *See Figures 4 (G, H-L) and S1 for quantification of migration. ^, % of neurons with mis-oriented leading processes (angle of leading process relative to the pial surface < 75º).

Table S8. Ciliopathy genes, neuronal identity and placement

Gene	Changes in % of GFP⁺/Tbr1⁺ neurons	Changes in % of GFP⁺/Ctip2⁺ neurons	Changes in % of GFP⁺/Cux1⁺ neurons	Changes in the laminar distribution of GFP⁺/Tbr1⁺ neurons	Changes in the laminar distribution of GFP⁺/Ctip2⁺ neurons	Changes in the laminar distribution of GFP⁺/Cux1⁺ neurons
AHI1	•	•	•	•	•	Yes (62 ± 2.9%)
ALMS1	•	•	•	•	•	Yes (79 ± 9.4%)
BBS1	•	•	•	•	•	Yes (29 ± 2.1%)
BBS4	•	•	•	•	•	Yes (34 ± 2.7%)
BBS9	•	•	•	•	•	Yes (24 ± 3.1%)
BBS10	•	•	•	•	•	Yes (39 ± 4.6%)
BBS11	•	•	•	•	•	Yes (15 ± 7.3%)
BBS12	•	•	•	•	•	Yes (21 ± 2.9%)
BUBR1	•	•	•	•	•	Yes (9.3 ± 3.6%)
IFT80	•	•	•	•	•	Yes (25 ± 5.2%)
KIF7	•	•	•	•	•	Yes (19 ± 3.2%)
NPHP1	•	•	•	•	•	Yes (59 ± 5.5%)
NPHP8	•	•	•	•	•	Yes (11 ± 2.3%)
TCTN2	•	•	•	•	•	Yes (6.3 ± 1.1%)
TMEM216	•	•	•	•	•	Yes (31 ± 2.9%)
TUB	•	•	•	•	•	Yes (17 ± 2.6 %)

No significant changes were observed in BBS2, BBS3, BBS5, BBS6, BBS7, BBS8, MKS1, MKS3, CEP290, CC2D2A, B9D1, OFD1, FTO, and CEP41 shRNA groups. Data shown are mean (± SEM). % of neurons in ectopic locations are indicated. Abbreviations used: •, no significant changes, p value ≥ 0.05, (Student's t test). Number of embryos/ group = 4. Number of total cells analyzed: GFP⁺/Tbr1⁺ [Control (602), AHI1 (453), ALMS1 (564), BBS1 (674), BBS4 (756), BBS7 (732), BBS9 (544), BBS10 (642), BBS11 (634), BBS12 (633), NPHP1 (547), NPHP8 (651), TCTN2 (622), TMEM216 (598), TUB (587)], GFP⁺/Ctip2⁺/Cux1⁺ [Control (606), AHI1 (467), ALMS1 (521), BBS1 (698), BBS4 (634), BBS7 (634), BBS9 (498), BBS10 (669), BBS11 (603), BBS12 (623), NPHP1 (587), NPHP8 (641), TCTN2 (702), TMEM216 (622, TUB (569)]. Also see Figure S2 for quantification.

Table S9. Ciliopathy genes and post-migratory neuronal differentiation

Gene	Changes in axonal outgrowth (fold basal)	Changes in midline crossing	Changes in axonal fasciculation	Changes in apical neurite morphology	Changes in apical neurite total length (μm) (fold basal)	Changes in # of apical neurite processes (fold basal)	Changes in # of basal neurite processes	Changes in filopodial density (fold basal)
BBS1	•	Yes	No	No	•	•	•	•
BBS5	↓ (0.64 ± 0.08)	Yes	Yes	No	•	•	•	•
BBS7	↓ (0.75 ± 0.02)	Yes	Yes	No	•	•	•	•
BBS9	•	No	Yes	Yes	↓ (0.33 ± 0.08)	↓ (0.467 ± 0.03)	•	↓ (0.39± 0.06)
BBS10	•	No	No	Yes	↓ (0.40 ± 0.02)	↓ (0.72 ± 0.02)	•	•
BBS11	•	Yes	Yes	Yes	↓ (0.65 ± 0.05)	↓ (0.68 ± 0.02)	•	↓ (0.51 ± 0.12)
BBS12	•	Yes	Yes	Yes	↓ (0.74 ± 0.03)	↓ (0.66 ± 0.03)	•	↓(0.28 ± 0.10)
KIF7	•	Yes	No	Yes	↓ (0.61 ± 0.09)	↓ (0.60 ± 0.03)	•	•
NPHP1	•	No	No	Yes	↓ (0.59 ± 0.06)	↓ (0.62 ± 0.03)	•	↓ (0.54 ± 0.09)
TMEM216	•	No	Yes	No	•	•	•	•

No significant changes were observed in ALMS1, AHI1, B9D1, BBS2, BBS3, BBS4, BBS6, BBS8, BUBR1, CC2D2A, CEP41, CEP290, FTO, IFT80, MKS1, MKS3, NPHP8, OFD1, TCTN2, and TUB shRNA groups. Data shown are mean (± SEM) fold basal changes. Abbreviation used: •, no significant changes; ↓, p value<0.05 (Student 's t test). Number of embryos/group = 4. Number of total cells analyzed: axonal outgrowth [Control (156), BBS5 (189), BBS7 (246)], number and length of neurites and filopodial density [Control (16), BBS9 (17), BBS10 (15), BBS11 (14), BBS12 (12), KIF7 (12), NPHP1 (19)]. Also see Figure 5 for quantification. Qualitative changes in midline crossing, axonal fasciculation were analyzed in serial sections obtained from 4 embryonic brains.

Table S10. Ciliopathy gene mutations associated with multiple aspects of human brain development

Gene	Neural tube patterning defects	Embryonic neurogenesis defects		Neuronal migration defects	Axonal projection defects (decussation)			Adult neurogenesis
	Encephalocele or exencephly	Microcephaly	Cerebellar hypoplasia	Polymicrogyria or Heterotopia or lissencephaly	CST	SCP	Corpus callosum	Hippocampal dysgenesis
AHI1			Yes[4,93]	Yes[94,95]	Yes[4,93]	Yes[4,93]	Yes[4,93]	
ALMS1*		Yes[25]					Yes[25]	
ARL13B	Yes[82]		Yes[82]	Yes[96]		Yes[82]		
BBS4*			Yes[83]		Yes[83]			
BUBR1*		Yes[26]	Yes[26]				Yes[26]	
C5orf42						Yes[50]	Yes[50]	
CC2D2A*			Yes[22]			Yes[22]	Yes[22]	
CENPF*		Yes[62]	Yes[62]				Yes[62]	
CEP41			Yes[5]			Yes[5]		
CEP120			Yes[56]			Yes[56]	Yes[56]	
CEP290			Yes[21]			Yes[21]		
CSPP1	Yes[41]		Yes[41]	Yes[41]		Yes[41]		
INPP5E			Yes[43]			Yes[43]		
KATNB1*		Yes[63]					Yes[63]	
KIF7*			Yes[31]			Yes[31]	Yes[31]	Yes[31]
MKS3*	Yes[85]		Yes[85]			Yes[85]		
NPHP1			Yes[86,87]			Yes[86,87]		
NPHP8	Yes[88,92]		Yes[88,92]			Yes[88,92]		
OFD1*			Yes[35]	Yes[35]		Yes[35]	Yes[35]	Yes[35]
RTTN*				Yes[81]			Yes[81]	
SCLT1		Yes[51]	Yes[51]	Yes[51]		Yes[51]	Yes[51]	
TBC1D32		Yes[51]	Yes[51]	Yes[51]		Yes[51]	Yes[51]	
TCTN2			Yes[45]			Yes[45]		
TMEM237*	Yes[89,90]		Yes[89,90]			Yes[89,90]		

Abbreviations used: CST, corticospinal tract; SCP, superior cerebellum peduncle; AHI1, Abelson helper integration site 1; ALMS1, Alstrom syndrome 1; ARL13B, ADP-ribosylation factor-like protein 13B; BBS4, Bardet-Biedl syndrome 4; BUBR1, Bub1-related kinase; C5orf42, chromosome 5 open reading frame 42; CC2D2A, coiled-coil and C2 domain containing 2A; CENPF, centromere protein F; CEP 41, centrosomal protein 41; CEP120, centrosomal protein 120; CEP 290, centrosomal protein 290; CSPP1, centrosome and spindle pole associated protein 1; INPP5E, Inositol polyphosphate-5-phosphatase; KATNB1, Katanin p80 subunit B1; KIF7, Kinesin family member 7; MKS3, Meckle Syndrome 3; NPHP, Nephronophthisis; OFD1, Oral-facial-digital syndrome 1; RTTN, rotatin; SCLT1, sodium channel and clathrin linker 1; TBC1D32, TBC1 domain family, member 32; TCTN2, tectonic family member 2; TMEM237, transmembrane protein 237. *, These gene mutations also cause hydrocephalus.

Supplemental References

1 Cheng, Y.-Z. *et al.* Investigating embryonic expression patterns and evolution of AHI1 and CEP290 genes, implicated in Joubert syndrome. *PloS one* **7**, e44975, doi:10.1371/journal.pone.0044975 (2012).

2 Lancaster, M. A. *et al.* Defective Wnt-dependent cerebellar midline fusion in a mouse model of Joubert syndrome. *Nature Medicine* **17**, 726-731, doi:10.1038/nm.2380 (2011).

3 Brancati, F., Dallapiccola, B. & Valente, E. M. Joubert Syndrome and related disorders. *Orphanet Journal of Rare Diseases* **5**, 20, doi:10.1186/1750-1172-5-20 (2010).

4 Dixon-Salazar, T. *et al.* Mutations in the AHI1 gene, encoding jouberin, cause Joubert syndrome with cortical polymicrogyria. *American journal of human genetics* **75**, 979-987, doi:10.1086/425985 (2004).

5 Lee, J. E. *et al.* CEP41 is mutated in Joubert syndrome and is required for tubulin glutamylation at the cilium. *Nature genetics* **44**, 193-199, doi:10.1038/ng.1078 (2012).

6 Nachury, M. V. *et al.* A core complex of BBS proteins cooperates with the GTPase Rab8 to promote ciliary membrane biogenesis. *Cell* **129**, 1201-1213, doi:10.1016/j.cell.2007.03.053 (2007).

7 Zhang, Q., Yu, D., Seo, S., Stone, E. M. & Sheffield, V. C. Intrinsic protein-protein interaction-mediated and chaperonin-assisted sequential assembly of stable bardet-biedl syndrome protein complex, the BBSome. *The Journal of Biological Chemistry* **287**, 20625-20635, doi:10.1074/jbc.M112.341487 (2012).

8 Soetedjo, L., Glover, D. v. A. & Jin, H. Targeting of vasoactive intestinal peptide receptor 2, VPAC2, a secretin family G-protein coupled receptor, to primary cilia. *Biology open* **2**, 686-694, doi:10.1242/bio.20134747 (2013).

9 Loktev, A. V. & Jackson, P. K. Neuropeptide Y Family Receptors Traffic via the Bardet-Biedl Syndrome Pathway to Signal in Neuronal Primary Cilia. *Cell Reports* **5**, 1316-1329, doi:10.1016/j.celrep.2013.11.011 (2013).

10 Rooryck, C. *et al.* Bardet-biedl syndrome and brain abnormalities. *NEUROPEDIATRICS* **38**, 5-9, doi:10.1055/s-2007-981466 (2007).

11 Zhang, Q. *et al.* Bardet-Biedl syndrome 3 (Bbs3) knockout mouse model reveals common BBS-associated phenotypes and Bbs3 unique phenotypes. *Proceedings of the National Academy of Sciences of the United States of America* **108**, 20678-20683, doi:10.1073/pnas.1113220108 (2011).

12 Seo, S. *et al.* BBS6, BBS10, and BBS12 form a complex with CCT/TRiC family chaperonins and mediate BBSome assembly. *Proceedings of the National Academy of Sciences of the United States of America* **107**, 1488-1493, doi:10.1073/pnas.0910268107 (2010).

13 Locke, M., Tinsley, C. L., Benson, M. A. & Blake, D. J. TRIM32 is an E3 ubiquitin ligase for dysbindin. *Human Molecular Genetics* **18**, 2344-2358, doi:10.1093/hmg/ddp167 (2009).

14 Garcia-Gonzalo, F. R. *et al.* A transition zone complex regulates mammalian ciliogenesis and ciliary membrane composition. *Nature genetics* **43**, 776-784, doi:10.1038/ng.891 (2011).

15 Williams, C. L. *et al.* MKS and NPHP modules cooperate to establish basal body/transition zone membrane associations and ciliary gate function during ciliogenesis. *The Journal of Cell Biology* **192**, 1023-1041, doi:10.1083/jcb.201012116 (2011).
16 Szymanska, K. & Johnson, C. A. The transition zone: an essential functional compartment of cilia. *Cilia* **1**, 10, doi:10.1186/2046-2530-1-10 (2012).
17 Valente, E. M. *et al.* Mutations in TMEM216 perturb ciliogenesis and cause Joubert, Meckel and related syndromes. *Nature genetics* **42**, 619-625, doi:10.1038/ng.594 (2010).
18 Leightner, A. C. *et al.* The Meckel syndrome protein meckelin (TMEM67) is a key regulator of cilia function but is not required for tissue planar polarity. *Human molecular genetics* **22**, 2024-2040, doi:10.1093/hmg/ddt054 (2013).
19 Abdelhamed, Z. A. *et al.* Variable expressivity of ciliopathy neurological phenotypes that encompass Meckel-Gruber syndrome and Joubert syndrome is caused by complex de-regulated ciliogenesis, Shh and Wnt signalling defects. *Human molecular genetics* **22**, 1358-1372, doi:10.1093/hmg/dds546 (2013).
20 Parisi, M. A. Clinical and molecular features of Joubert syndrome and related disorders. *American journal of medical genetics Part C, Seminars in medical genetics* **151C**, 326-340, doi:10.1002/ajmg.c.30229 (2009).
21 Valente, E. M. *et al.* Mutations in CEP290, which encodes a centrosomal protein, cause pleiotropic forms of Joubert syndrome. *Nature Genetics* **38**, 623-625, doi:10.1038/ng1805 (2006).
22 Gorden, N. T. *et al.* CC2D2A is mutated in Joubert syndrome and interacts with the ciliopathy-associated basal body protein CEP290. *American journal of human genetics* **83**, 559-571, doi:10.1016/j.ajhg.2008.10.002 (2008).
23 Jagger, D. *et al.* Alström Syndrome protein ALMS1 localizes to basal bodies of cochlear hair cells and regulates cilium-dependent planar cell polarity. *Human Molecular Genetics* **20**, 466-481, doi:10.1093/hmg/ddq493 (2011).
24 Heydet, D. *et al.* A truncating mutation of Alms1 reduces the number of hypothalamic neuronal cilia in obese mice. *Developmental Neurobiology* **73**, 1-13, doi:10.1002/dneu.22031 (2013).
25 Citton, V. *et al.* Brain involvement in Alström syndrome. *Orphanet Journal of Rare Diseases* **8**, 24-24, doi:info:pmid/23406482 (2013).
26 Miyamoto, T. *et al.* Insufficiency of BUBR1, a mitotic spindle checkpoint regulator, causes impaired ciliogenesis in vertebrates. *Human Molecular Genetics* **20**, 2058-2070, doi:10.1093/hmg/ddr090 (2011).
27 Suijkerbuijk, S. J. E. *et al.* Molecular causes for BUBR1 dysfunction in the human cancer predisposition syndrome mosaic variegated aneuploidy. *Cancer Research* **70**, 4891-4900, doi:10.1158/0008-5472.CAN-09-4319 (2010).
28 Kajii, T. *et al.* Cancer-prone syndrome of mosaic variegated aneuploidy and total premature chromatid separation: report of five infants. *American journal of medical genetics* **104**, 57-64 (2001).
29 Rix, S., Calmont, A., Scambler, P. J. & Beales, P. L. An Ift80 mouse model of short rib polydactyly syndromes shows defects in hedgehog signalling without loss or malformation of cilia. *Human molecular genetics* **20**, 1306-1314, doi:10.1093/hmg/ddr013 (2011).

30 Lehman, A. M. *et al.* Co-Occurrence of Joubert Syndrome and Jeune Asphyxiating Thoracic Dystrophy. *American journal of medical genetics. Part A* **0**, 1411, doi:10.1002/ajmg.a.33416.
31 Putoux, A. *et al.* KIF7 mutations cause fetal hydrolethalus and acrocallosal syndromes. *Nature Genetics* **43**, 601-606, doi:10.1038/ng.826 (2011).
32 Liem, K. F., He, M., Ocbina, P. J. R. & Anderson, K. V. Mouse Kif7/Costal2 is a cilia-associated protein that regulates Sonic hedgehog signaling. *Proceedings of the National Academy of Sciences of the United States of America* **106**, 13377-13382, doi:10.1073/pnas.0906944106 (2009).
33 Singla, V., Romaguera-Ros, M., Garcia-Verdugo, J. M. & Reiter, J. F. Ofd1, a human disease gene, regulates the length and distal structure of centrioles. *Developmental cell* **18**, 410-424, doi:10.1016/j.devcel.2009.12.022 (2010).
34 Macca, M. & Franco, B. The molecular basis of oral-facial-digital syndrome, type 1. *American journal of medical genetics Part C, Seminars in medical genetics* **151C**, 318-325, doi:10.1002/ajmg.c.30224 (2009).
35 Bisschoff, I. J. *et al.* Novel mutations including deletions of the entire OFD1 gene in 30 families with type 1 orofaciodigital syndrome: a study of the extensive clinical variability. *Human mutation* **34**, 237-247, doi:10.1002/humu.22224 (2013).
36 Mukhopadhyay, S. & Jackson, P. K. The tubby family proteins. *Genome Biology* **12**, 225, doi:10.1186/gb-2011-12-6-225 (2011).
37 Sun, X. *et al.* Tubby is required for trafficking G protein-coupled receptors to neuronal cilia. *Cilia* **1**, 21, doi:10.1186/2046-2530-1-21 (2012).
38 Ho, A. J. *et al.* A commonly carried allele of the obesity-related FTO gene is associated with reduced brain volume in the healthy elderly. *Proceedings of the National Academy of Sciences of the United States of America* **107**, 8404-8409, doi:10.1073/pnas.0910878107 (2010).
39 Osborn, D. P. S. *et al.* Loss of FTO antagonises Wnt signaling and leads to developmental defects associated with ciliopathies. *PloS one* **9**, e87662, doi:10.1371/journal.pone.0087662 (2014).
40 Larkins, C. E., Aviles, G. D. G., East, M. P., Kahn, R. A. & Caspary, T. Arl13b regulates ciliogenesis and the dynamic localization of Shh signaling proteins. *Molecular Biology of the Cell* **22**, 4694-4703, doi:10.1091/mbc.E10-12-0994 (2011).
41 Tuz, K. *et al.* Mutations in CSPP1 cause primary cilia abnormalities and Joubert syndrome with or without Jeune asphyxiating thoracic dystrophy. *American journal of human genetics* **94**, 62-72, doi:10.1016/j.ajhg.2013.11.019 (2014).
42 Lee, J. H. *et al.* Evolutionarily assembled cis-regulatory module at a human ciliopathy locus. *Science* **335**, 966-969, doi:10.1126/science.1213506 (2012).
43 Jacoby, M. *et al.* INPP5E mutations cause primary cilium signaling defects, ciliary instability and ciliopathies in human and mouse. *Nature genetics* **41**, 1027-1031, doi:10.1038/ng.427 (2009).
44 Apostolou, T. *et al.* Late onset of renal disease in nephronophthisis with features of Joubert syndrome type B. *Nephrology, dialysis, transplantation : official publication of the European Dialysis and Transplant Association - European Renal Association* **16**, 2412-2415 (2001).

45 Sang, L. *et al.* Mapping the NPHP-JBTS-MKS protein network reveals ciliopathy disease genes and pathways. *Cell* **145**, 513-528, doi:10.1016/j.cell.2011.04.019 (2011).
46 Barbelanne, M., Song, J., Ahmadzai, M. & Tsang, W. Y. Pathogenic NPHP5 mutations impair protein interaction with Cep290, a prerequisite for ciliogenesis. *Human Molecular Genetics* **22**, 2482-2494, doi:10.1093/hmg/ddt100 (2013).
47 Ramachandran, H. *et al.* Interaction with the Bardet-Biedl Gene Product TRIM32/BBS11 Modifies the Half-life and Localization of Glis2/NPHP7. *Journal of Biological Chemistry* **289**, 8390-8401, doi:10.1074/jbc.M113.534024 (2014).
48 Fukui, H., Shiba, D., Asakawa, K., Kawakami, K. & Yokoyama, T. The ciliary protein Nek8/Nphp9 acts downstream of Inv/Nphp2 during pronephros morphogenesis and left-right establishment in zebrafish. *FEBS letters* **586**, 2273-2279, doi:10.1016/j.febslet.2012.05.064 (2012).
49 Failler, M. *et al.* Mutations of CEP83 cause infantile nephronophthisis and intellectual disability. *American journal of human genetics* **94**, 905-914, doi:10.1016/j.ajhg.2014.05.002 (2014).
50 Bayram, Y. *et al.* Exome sequencing identifies a homozygous C5orf42 variant in a Turkish kindred with oral-facial-digital syndrome type VI. *American Journal of Medical Genetics Part A*, doi:10.1002/ajmg.a.37092 (2015).
51 Adly, N., Alhashem, A., Ammari, A. & Alkuraya, F. S. Ciliary genes TBC1D32/C6orf170 and SCLT1 are mutated in patients with OFD type IX. *Human Mutation* **35**, 36-40, doi:10.1002/humu.22477 (2014).
52 Nauli, S. M. *et al.* Polycystins 1 and 2 mediate mechanosensation in the primary cilium of kidney cells. *Nature Genetics* **33**, 129-137, doi:10.1038/ng1076 (2003).
53 Yoder, B. K. Role of primary cilia in the pathogenesis of polycystic kidney disease. *Journal of the American Society of Nephrology : JASN* **18**, 1381-1388, doi:10.1681/ASN.2006111215 (2007).
54 Ibañez-Tallon, I., Gorokhova, S. & Heintz, N. Loss of function of axonemal dynein Mdnah5 causes primary ciliary dyskinesia and hydrocephalus. *Human Molecular Genetics* **11**, 715-721 (2002).
55 Ostrowski, L. E. *et al.* Conditional deletion of dnaic1 in a murine model of primary ciliary dyskinesia causes chronic rhinosinusitis. *American Journal of Respiratory Cell and Molecular Biology* **43**, 55-63, doi:10.1165/rcmb.2009-0118OC (2010).
56 Shaheen, R. *et al.* A founder CEP120 mutation in Jeune asphyxiating thoracic dystrophy expands the role of centriolar proteins in skeletal ciliopathies. *Human molecular genetics* **24**, 1410-1419, doi:10.1093/hmg/ddu555 (2015).
57 al-Gazali, L. I. *et al.* Complex consanguinity associated with short rib-polydactyly syndrome III and congenital infection-like syndrome: a diagnostic problem in dysmorphic syndromes. *Journal of Medical Genetics* **36**, 461-466 (1999).
58 El Hokayem, J. *et al.* NEK1 and DYNC2H1 are both involved in short rib polydactyly Majewski type but not in Beemer Langer cases. *Journal of Medical Genetics* **49**, 227-233, doi:10.1136/jmedgenet-2011-100717 (2012).
59 Shalom, O., Shalva, N., Altschuler, Y. & Motro, B. The mammalian Nek1 kinase is involved in primary cilium formation. *FEBS letters* **582**, 1465-1470, doi:10.1016/j.febslet.2008.03.036 (2008).

60 Dagoneau, N. *et al.* DYNC2H1 mutations cause asphyxiating thoracic dystrophy and short rib-polydactyly syndrome, type III. *American journal of human genetics* **84**, 706-711, doi:10.1016/j.ajhg.2009.04.016 (2009).
61 Davis, E. E. *et al.* TTC21B contributes both causal and modifying alleles across the ciliopathy spectrum. *Nature Genetics* **43**, 189-196, doi:10.1038/ng.756 (2011).
62 Waters, A. M. *et al.* The kinetochore protein, CENPF, is mutated in human ciliopathy and microcephaly phenotypes. *Journal of Medical Genetics* **52**, 147-156, doi:10.1136/jmedgenet-2014-102691 (2015).
63 Hu, W. F. *et al.* Katanin p80 regulates human cortical development by limiting centriole and cilia number. *Neuron* **84**, 1240-1257, doi:10.1016/j.neuron.2014.12.017 (2014).
64 Mcintyre, J. C. *et al.* Gene therapy rescues cilia defects and restores olfactory function in a mammalian ciliopathy model. *Nature Medicine* **18**, 1423-1428, doi:10.1038/nm.2860 (2012).
65 den Hollander, A. I., Roepman, R., Koenekoop, R. K. & Cremers, F. P. M. Leber congenital amaurosis: genes, proteins and disease mechanisms. *Progress in retinal and eye research* **27**, 391-419, doi:10.1016/j.preteyeres.2008.05.003 (2008).
66 Mackay, D. S. *et al.* Screening of a large cohort of leber congenital amaurosis and retinitis pigmentosa patients identifies novel LCA5 mutations and new genotype-phenotype correlations. *Human mutation* **34**, 1537-1546, doi:10.1002/humu.22398 (2013).
67 Kanan, Y., Wicker, L. D., Al-Ubaidi, M. R., Mandal, N. A. & Kasus-Jacobi, A. Retinol dehydrogenases RDH11 and RDH12 in the mouse retina: expression levels during development and regulation by oxidative stress. *Investigative Ophthalmology & Visual Science* **49**, 1071-1078, doi:10.1167/iovs.07-1207 (2008).
68 Kennan, A. *et al.* Identification of an IMPDH1 mutation in autosomal dominant retinitis pigmentosa (RP10) revealed following comparative microarray analysis of transcripts derived from retinas of wild-type and Rho(-/-) mice. *Human Molecular Genetics* **11**, 547-557 (2002).
69 Yang, H. K., Hwang, J.-M., Park, S. S. & Yu, Y. S. Brain imaging studies in Leber's congenital amaurosis: new radiologic findings associated with the complex trait. *Korean Journal of Ophthalmology* **24**, 360-363, doi:10.3341/kjo.2010.24.6.360 (2010).
70 Freund, C. L. *et al.* De novo mutations in the CRX homeobox gene associated with Leber congenital amaurosis. *Nature genetics* **18**, 311-312, doi:10.1038/ng0498-311 (1998).
71 Roosing, S. *et al.* Maternal uniparental isodisomy of chromosome 6 reveals a TULP1 mutation as a novel cause of cone dysfunction. *Ophthalmology* **120**, 1239-1246, doi:10.1016/j.ophtha.2012.12.005 (2013).
72 Sergouniotis, P. I. *et al.* Recessive mutations in KCNJ13, encoding an inwardly rectifying potassium channel subunit, cause leber congenital amaurosis. *American Journal of Human Genetics* **89**, 183-190, doi:10.1016/j.ajhg.2011.06.002 (2011).

73 den Hollander, A. I. *et al.* Leber congenital amaurosis and retinitis pigmentosa with Coats-like exudative vasculopathy are associated with mutations in the crumbs homologue 1 (CRB1) gene. *American Journal of Human Genetics* **69**, 198-203 (2001).
74 Perrault, I. *et al.* Spectrum of SPATA7 mutations in Leber congenital amaurosis and delineation of the associated phenotype. *Human mutation* **31**, E1241-1250, doi:10.1002/humu.21203 (2010).
75 Beck, B. B. *et al.* Mutation of POC1B in a severe syndromic retinal ciliopathy. *Human Mutation* **35**, 1153-1162, doi:10.1002/humu.22618 (2014).
76 Bujakowska, K. M. *et al.* Mutations in IFT172 cause isolated retinal degeneration and Bardet-Biedl syndrome. *Human Molecular Genetics* **24**, 230-242, doi:10.1093/hmg/ddu441 (2015).
77 Aldahmesh, M. A. *et al.* IFT27, encoding a small GTPase component of IFT particles, is mutated in a consanguineous family with Bardet-Biedl syndrome. *Human Molecular Genetics* **23**, 3307-3315, doi:10.1093/hmg/ddu044 (2014).
78 Scheidecker, S. *et al.* Exome sequencing of Bardet-Biedl syndrome patient identifies a null mutation in the BBSome subunit BBIP1 (BBS18). *Journal of Medical Genetics* **51**, 132-136, doi:10.1136/jmedgenet-2013-101785 (2014).
79 Filges, I. *et al.* Exome sequencing identifies mutations in KIF14 as a novel cause of an autosomal recessive lethal fetal ciliopathy phenotype. *Clinical genetics* **86**, 220-228, doi:10.1111/cge.12301 (2014).
80 Blackburn, M. G. & Belliveau, R. E. Ellis-van Creveld syndrome. A report of previously undescribed anomalies in two siblings. *American journal of diseases of children (1960)* **122**, 267-270 (1971).
81 Kheradmand Kia, S. *et al.* RTTN mutations link primary cilia function to organization of the human cerebral cortex. *American Journal of Human Genetics* **91**, 533-540, doi:10.1016/j.ajhg.2012.07.008 (2012).
82 Cantagrel, V. *et al.* Mutations in the cilia gene ARL13B lead to the classical form of Joubert syndrome. *American Journal of Human Genetics* **83**, 170-179, doi:10.1016/j.ajhg.2008.06.023 (2008).
83 Karmous-Benailly, H. *et al.* Antenatal presentation of Bardet-Biedl syndrome may mimic Meckel syndrome. *American journal of human genetics* **76**, 493-504, doi:10.1086/428679 (2005).
84 Bielas, S. L. *et al.* Mutations in INPP5E, encoding inositol polyphosphate-5-phosphatase E, link phosphatidyl inositol signaling to the ciliopathies. *Nature genetics* **41**, 1032-1036, doi:10.1038/ng.423 (2009).
85 Brancati, F. *et al.* MKS3/TMEM67 mutations are a major cause of COACH Syndrome, a Joubert Syndrome related disorder with liver involvement. *Human mutation* **30**, E432-442, doi:10.1002/humu.20924 (2009).
86 Parisi, M. A. *et al.* The NPHP1 gene deletion associated with juvenile nephronophthisis is present in a subset of individuals with Joubert syndrome. *American journal of human genetics* **75**, 82-91, doi:10.1086/421846 (2004).
87 Castori, M. *et al.* NPHP1 gene deletion is a rare cause of Joubert syndrome related disorders. *Journal of Medical Genetics* **42**, e9, doi:10.1136/jmg.2004.027375 (2005).

88 Delous, M. *et al.* The ciliary gene RPGRIP1L is mutated in cerebello-oculo-renal syndrome (Joubert syndrome type B) and Meckel syndrome. *Nature genetics* **39**, 875-881, doi:10.1038/ng2039 (2007).
89 Huang, L. *et al.* TMEM237 is mutated in individuals with a Joubert syndrome related disorder and expands the role of the TMEM family at the ciliary transition zone. *American Journal of Human Genetics* **89**, 713-730, doi:10.1016/j.ajhg.2011.11.005 (2011).
90 Boycott, K. M. *et al.* Meckel syndrome in the Hutterite population is actually a Joubert-related cerebello-oculo-renal syndrome. *American Journal of Medical Genetics Part A* **143A**, 1715-1725, doi:10.1002/ajmg.a.31832 (2007).
91 Forsythe, E. & Beales, P. L. Bardet-Biedl syndrome. *European journal of human genetics : EJHG* **21**, 8-13, doi:10.1038/ejhg.2012.115 (2013).
92 Arts, H. H. *et al.* Mutations in the gene encoding the basal body protein RPGRIP1L, a nephrocystin-4 interactor, cause Joubert syndrome. *Nature genetics* **39**, 882-888, doi:10.1038/ng2069 (2007).
93 Giordano, L. *et al.* Joubert syndrome with bilateral polymicrogyria: clinical and neuropathological findings in two brothers. *American journal of medical genetics Part A* **149A**, 1511-1515, doi:10.1002/ajmg.a.32936 (2009).
94 Simms, R. J. *et al.* Modelling a ciliopathy: Ahi1 knockdown in model systems reveals an essential role in brain, retinal, and renal development. *Cellular and molecular life sciences : CMLS* **69**, 993-1009, doi:10.1007/s00018-011-0826-z (2012).
95 Valente, E. M. *et al.* AHI1 gene mutations cause specific forms of Joubert syndrome-related disorders. *Annals of neurology* **59**, 527-534, doi:10.1002/ana.20749 (2006).
96 Higginbotham, H. *et al.* Arl13b in primary cilia regulates the migration and placement of interneurons in the developing cerebral cortex. *Developmental Cell* **23**, 925-938, doi:10.1016/j.devcel.2012.09.019 (2012).
97 Thomas, S. *et al.* Identification of a novel ARL13B variant in a Joubert syndrome-affected patient with retinal impairment and obesity. *European journal of human genetics* **23**, 621-627, doi:10.1038/ejhg.2014.156 (2014).
98 Diez-Roux, G. *et al.* A high-resolution anatomical atlas of the transcriptome in the mouse embryo. *PLOS Biology* **9**, e1000582, doi:10.1371/journal.pbio.1000582 (2011).
99 Thompson, C. L. *et al.* A High-Resolution Spatiotemporal Atlas of Gene Expression of the Developing Mouse Brain. *Neuron*, doi:10.1016/j.neuron.2014.05.033 (2014).
100 Kang, H. J. *et al.* Spatio-temporal transcriptome of the human brain. *Nature* **478**, 483-489, doi:10.1038/nature10523 (2011).
101 Knuesel, I. *et al.* Maternal immune activation and abnormal brain development across CNS disorders. *Nature reviews Neurology* **10**, 643-660, doi:10.1038/nrneurol.2014.187 (2014).
102 Stiles, J. & Jernigan, T. L. The basics of brain development. *Neuropsycology Reviews* **20**, 327-348, doi:10.1007/s11065-010-9148-4 (2010).
